# A putative lateral flagella of the cystic fibrosis pathogen *Burkholderia dolosa* regulates swimming motility and host cytokine production

**DOI:** 10.1371/journal.pone.0189810

**Published:** 2018-01-18

**Authors:** Damien Roux, Matthew Schaefers, Bradley S. Clark, Molly Weatherholt, Diane Renaud, David Scott, John J. LiPuma, Gregory Priebe, Craig Gerard, Deborah R. Yoder-Himes

**Affiliations:** 1 Department of Biology, University of Louisville, Louisville, Kentucky, United States of America; 2 INSERM, IAME, UMR 1137, Paris, France; 3 Univ Paris Diderot, Sorbonne Paris Cité, Paris, France; 4 AP-HP, Louis Mourier Hospital, Intensive Care Unit, Colombes, France; 5 Division of Critical Care Medicine, Department of Anesthesiology, Perioperative and Pain Medicine, Boston Children’s Hospital, Boston, Massachusetts, United States of America; 6 Department of Anesthesia, Harvard Medical School, Boston, Massachusetts, United States of America; 7 Oral Immunology and Infectious Diseases, School of Dentistry, University of Louisville, Louisville, Kentucky, United States of America; 8 Division of Pediatrics, University of Michigan, Ann Arbor, Michigan, United States of America; 9 Division of Respiratory Diseases, Boston Children’s Hospital, Boston, Massachusetts, United States of America; Laurentian, CANADA

## Abstract

*Burkholderia dolosa* caused an outbreak in the cystic fibrosis clinic at Boston Children’s Hospital and was associated with high mortality in these patients. This species is part of a larger complex of opportunistic pathogens known as the *Burkholderia cepacia* complex (Bcc). Compared to other species in the Bcc, *B*. *dolosa* is highly transmissible; thus understanding its virulence mechanisms is important for preventing future outbreaks. The genome of one of the outbreak strains, AU0158, revealed a homolog of the *lafA* gene encoding a putative lateral flagellin, which, in other non-Bcc species, is used for movement on solid surfaces, attachment to host cells, or movement inside host cells. Here, we analyzed the conservation of the *lafA* gene and protein sequences, which are distinct from those of the polar flagella, and found *lafA* homologs to be present in numerous β-proteobacteria but notably absent from most other Bcc species. A *lafA* deletion mutant in *B*. *dolosa* showed a greater swimming motility than wild-type due to an increase in the number of polar flagella, but did not appear to contribute to biofilm formation, host cell invasion, or murine lung colonization or persistence over time. However, the *lafA* gene was important for cytokine production in human peripheral blood mononuclear cells, suggesting it may have a role in recognition by the human immune response.

## Introduction

Cystic fibrosis (CF) has a prevalence of ~1/3,000 live Caucasian births, making it a commonly inherited disorder leading to a limited lifespan and high morbidity [1]. Mutations within the chloride ion transporter gene, *CFTR*, result in CF and lead to the production of a thick, sticky, low pH mucus in the lungs of CF patients, creating a breeding ground for bacteria (reviewed in [2]). Common pathogens infecting the lungs of CF patients [3] include *Pseudomonas aeruginosa*, *Staphylococcus aureus*, *Haemophilus influenzae*, *Stenotrophomonas maltophilia*, and members of the Gram negative *Burkholderia cepacia* complex (Bcc), which is comprised of over 20 species closely related species including the intracellular opportunist *B*. *dolosa* [4].

Members of the Bcc are capable of producing a necrotizing pneumonia in infected patients, characterized by a rapid decline in lung function (1–3 months) with bacteremia and near 100% mortality. This is often called the “cepacia syndrome” [5]. Certain species within the Bcc are capable of facile patient-to-patient transmission and have caused outbreaks in CF clinics worldwide [6–8]. The most common outbreak strains capable of spreading between CF patients fall within the *B*. *cenocepacia* and *B*. *dolosa* species [9]. These outbreaks have resulted in the enforcement of strict regulations and segregation for CF patients. One of the outbreaks occurred at Boston Children’s Hospital, in which *B*. *dolosa* infected over 40 patients and contributed to the death of many, if not all, of them [10].

Genomic research on the Bcc has revealed that these bacteria have large genomes, consisting of multiple replicons. *B*. *dolosa* strain AU1058, one of the outbreak isolates, has a 6.41 Mb genome consisting of 66.81% GC pairs [11] organized in two circular chromosomes and a third chromosome/megaplasmid similar to other Bcc species [12–18]. Virulence traits attributed to *B*. *dolosa* and other members of the Bcc include intrinsic antimicrobial resistance (with some strains of *B*. *dolosa* and *B*. *cenocepacia* being nearly pan-resistant) [19–21], oxygen scavenging proteins [22], flagella [23, 24], biofilm formation [25, 26], LPS [27, 28], quorum sensing [29–33], siderophore production [34–37], oxygen-sensing transcriptional regulators [38], invasion and intracellular survival within macrophages for up to 5 days [39–41], and Type III Secretion Systems [42–44]. Based on these data, in conjunction with the observed pathogenicity of *B*. *dolosa* and other Bcc members in CF patients, it is crucial that the mechanism(s) by which these species interact with their hosts be understood before new effective therapeutics can be discovered to combat them.

The genome of the AU0158 outbreak isolate of *B*. *dolosa* was found to contain genes encoding both a polar flagellum (*fliC*) as well as an additional flagellin gene located at the AK34_RS07895 locus. Preliminary evidence suggests that the protein encoded at AK34_RS07895 serves as the monomer for lateral flagella which are found infrequently in bacteria but contribute to movement on solid surfaces in other pathogens such as *Aeromonas hydrophila* and *Vibrio parahaemolyticus* [45, 46]. These organisms often express the polar flagellum alone while grown in liquid culture, but express both polar and lateral flagella when grown on a solid medium. The polar flagellum of *A*. *hydrophila* has also been shown to act as a sensor that regulates the expression of the lateral flagellin genes [47]. Additionally, it was found that this polar-flagellum-induced increase in lateral flagella gene expression resulted in an increased formation of linkages between bacterial cells that contributed to microcolony and subsequent biofilm formation [48, 49]. Our group has previously explored the host immune response to *B*. *dolosa* and examined the role of polar flagellum in infection [50] in which we found that wild-type *B*. *dolosa* AU0158 is not recognized by the host immune response *in vivo* in murine models but it is recognized *in vitro* by cultured cells. Further, the polar flagella may have a role in infection but this may be masked *in vivo*. The goal of this study was to examine the gene encoding the putative lateral flagellin in *B*. *dolosa* in terms of evolutionary conservation and to determine if this putative lateral flagellin gene influenced motility, biofilm formation, host cell invasion, host lung colonization, and host cytokine production in response to *B*. *dolosa*.

## Materials and methods

### Bacterial cultures, human, and murine cell lines

*E*. *coli* DH5α and SM10λpir were used for cloning procedures. *B*. *dolosa* strains AU13297 (CF sputum isolate), AU0746 (CF sputum isolate), AU3960 (non-CF hemolytic disorder isolate), AU6240 (CF sputum isolate), AU6423 (CF sputum isolate), AU9628 (CF sputum isolate), and AY14895 (non-CF tracheal aspirate isolate) were obtained from the U.S. *Burkholderia cepacia* Research Laboratory and Repository. *P*. *aeruginosa* PAO1, a standard lab strain, was provided by Stephen Lory. All bacterial species were maintained in Lysogeny (Luria) Broth (LB) Lennox formulation unless otherwise noted. Kanamycin (50 μg/ml or 75 μg/ml), gentamicin (15 μg/ml), ampicillin (100 μg/ml), tetracycline (10 μg/ml or 75 μg/ml), irgasan (25 μg/ml), or 10% sucrose were added as indicated. Human peripheral blood mononuclear cells (PBMCs) were isolated from whole blood obtained from anonymized healthy donors. Cell lines were maintained at 5% CO_2_. The murine macrophage cell line RAW264.7 was cultured in Dulbecco’s Modified Eagle Medium (DMEM) with 10% FBS.

### Conservation bioinformatics and PCR

For each *B*. *dolosa* AU0158 flagella-associated gene, BLAST searches of the associated amino acid sequences was conducted in both the burkholderia.com database [15] and GenBank. For the majority of genes, two homologs were present (<1e^-10^) with one homolog being more similar to polar flagella system proteins and the other homolog more similar to lateral flagella homologs. The threshold used to determine a homolog changed dynamically with each gene but in general, there was a wide discrepancy in e-values allowing for assignment as more likely polar flagella-related or lateral flagella-related as these systems are quite distinct from each other at the gene and protein level.

The protein sequence of the *B*. *dolosa* AU0158 *lafA* gene was obtained from http://www.burkholderia.com [15] and used to BLAST [51] two databases: the burkholderia.com collection (for other *Burkholderia* species); and the nr database at Genbank [52]. The closest homologs in other species were extracted and aligned with Clustal Omega [53]. For visualization, an.aln file of the resultant alignment was uploaded into ESPript [54] using default parameters. For phylogenetic network construction, a.nxs file of the resultant alignment was uploaded into the Splitstree program [55].

To experimentally determine whether the *lafA* gene is conserved in all strains, genomic DNA was extracted from outbreak and non-outbreak *B*. *dolosa* isolates using the method described in [56] except that 0.5 mL of overnight culture was used and 3 μL of RNaseA (10 mg/ml) was added during the lysis step. PCR was performed on 200 ng genomic DNA using GoTaq Green Master Mix (Promega) and 10 μM each Laf_OE_up and Laf_OE_down (Table 1) which should produce a band of 889 bp.

**Table 1:** Primers used in this study.

Primer name	Sequence
Laf_OE_up	5’-ATATCATATGCACCACCACCACCACCACATGGCAATGAGC GTACATACCAACTCGG-3’
Laf_OE_down	5’-ATATCTCGAGTTACTGGACCAGAGACAGCACCATCTG-3’
FliC RT_PCR-up	5'- CACGACGACGCAGACGCAGG -3'
FliC RT_PCR-down	5'- GCCTTGCTCAGGTTCGCCG -3'
LafA RT_PCR-up	5'- GATGGCAGTCCGTCTGGAGGC -3'
LafA RT_PCR-down	5'- GATACAGGATGTTCGTCGCTTCGC -3'
RpoD_up_RT	5'- TCTGCTTCTTCCGCCAGGACG -3'
RpoD_down_RT	5'- GCACTTGCCCGCTTCGCC -3
Bd_lafA_Dl-5’up	5'-TTTTTGCGGCCGCTCGGCAGCGAGACCTGCTTGC-3'
Bd_lafA_Dl-5’down	5'-CAGGCGACCGGCGCCGGCCGTTTCAAACTCCATTGGTCGTGG TCATCC-3'
Bd_lafA_Dl-3’up	5'-GGATGACCACGACCAATGGAGTTTGAAACGGCCGGCGCCG GTC GCCTG-3'
Bd_lafA_Dl-3’down	5'-TTTTTGAATTCCTGTCGGCCTCGAGGCGCTCG-3'
*lafA* (AK34_4737) Forward	5’-GATTCGACGGAACATGCAAAG-3’
*lafA* (AK34_4737) Reverse	5’-CATTGTCCTTGGCCGAATTG-3’
LafAexp-UP	5’-ATATATGAATTCGGTCGGTCGGGGTGGCAAG-3’
LafAexp_DOWN	5’-ATATATCCCGGGGTCACGACAACCCTGAACGAATCCTGAACG-3’

To visualize regions of conservation surrounding the *lafA* gene in other *Burkholderia* species, precomputed alignments were analyzed for conservation surround AK34_RS07895 in the VISTA browser located at Integrated Microbial Genomes website: (https://img.jgi.doe.gov/cgi-bin/w/main.cgi?section=Vista&page=vista). Default parameters for conservation were used. Microbial genomes chosen for comparison included multiple *B*. *cenocepacia*, *B*. *multivorans*, and *B*. *ambifaria* strains as well as select single strains/isolates of other *Burkholderia* species.

### *B*. *dolosa* mutant generation

An in-frame deletion mutation in the *B*. *dolosa lafA* gene was generated via gene splicing by overlap extension [57] as previously described {Roux, 2017 #7359}. Briefly, 500 bp upstream and 500 bp downstream of AK34_RS07895 (sequences obtained at http://www.burkholderia.com [15]) was amplified by PCR using Bd_lafA_Dl-5’up and Bd_lafA_Dl-3’up or Bd_lafA_Dl-5’down and Bd_lafA_Dl-3’down (Table 1) respectively. These templates were purified and used in a second PCR as templates with Bd_lafA_Dl-5’up and Bd_lafA_Dl-3’down to generate fused amplicons. These amplicons were cut using the *Xma*I and *Eco*RI restriction enzymes and ligated into in the *Xma*I and *Eco*RI sites of pEXKm5 [58] to generate the pEXKm5Tet-lafAdel plasmid. They were then transformed into DH5α competent cells in LB agar supplemented with 50 μg/ml kanamycin, followed by Sanger sequencing. Cells of the conjugative donor *E*. *coli* strain SM10λpir were transformed with pEXKm5Tet-lafAdel and selected on LB agar supplemented with 50 μg/ml kanamycin + 10 μg/ml tetracycline. Plasmids were then transferred into *B*. *dolosa* by conjugation and insertion of the plasmid into the chromosome was verified by PCR. Counter-selection was performed by plating the verified transconjugants on LB medium + 10% sucrose for *B*. *dolosa* and checked for loss of the plasmid backbone by blue-white screening on X-Gluc (25 μg/ml)-containing LB medium and checked for *lafA* gene deletion by PCR. The growth rates of mutant strains were not significantly different than the wild-type in LB liquid medium.

To generate the *lafA* complemented strain, PCR was used to amplify 253 bp upstream of BDAG_04366 through the coding region with the LafAexp-UP and LafAexp_DOWN primers. The amplicon was cloned into pUCP18T-miniTn7-Tp as a *Xma*I/*Eco*RI fragment and verified by Sanger sequencing to generate pUCP18T-miniTn7-Tp-*lafA*. *B*. *dolosa* AU1058 wild-type, Δ*fliC*, and Δ*lafA* were conjugated with *E*. *coli* SM10 λpir containing pUCP18T-miniTn7-Tp empty vector and transconjugants were selected on LB + 1000 μg/ml trimethoprim + 10 μg/ml gentamicin. In parallel, *B*. *dolosa* Δ*lafA* was also conjugated with pUCP18T-miniTn7-Tp-*lafA*. Transconjugants were verified by PCR for transposon insertion downstream of BDAG_04221 and to verify the original flagellin deletions where appropriate.

### Motility assays

LB agar plates containing 0.2%, 0.3%, 0.4%, or 0.5% agar were prepared by pipetting 30 mL of molten agar into sterile petri plates and allowed to solidify for 4 hours. Mid-log phase cultures of *B*. *dolosa* strain AU0158 wild-type containing the pUCP18T-miniTn7-Tp empty vector, an AU0158 *fliC* deletion strain created previously {Roux, 2017 #7359} containing the pUCP18T-miniTn7-Tp empty vector, the AU0158 *lafA* mutant strain containing the pUCP18T-miniTn7-Tp empty vector, and the AU0158 *lafA* mutant strain containing the pUCP18T-miniTn7-Tp-*lafA* complementing vector were normalized to O.D._600_ of 1.0 and ten microliter aliquots were spotted on LB plates containing 0.2%, 0.3%, 0.4%, or 0.5% agar (Alfa Aesar, Cat#10752–36) either individually or together on plates. After 1 hour of drying to ensure the culture liquid was absorbed, the plates were incubated at 37°C right-side up. After 24–48 hours, the diameters of the resulting spots were measured. The average diameter of *B*. *dolosa* AU0158 + empty vector replicates for a given experiment was used to normalize all diameters to account for different plates and for separate experiments. Four to twenty-seven biological and technical replicates were run and significant differences were established by one-way and two-way ANOVAs with Tukey’s multiple testing correction.

### Microscopy

For examination of flagella by transmission electron microscopy (TEM) *B*. *dolosa* AU0158 wild-type, Δ*fliC*, and Δ*lafA* mutant strains were grown for 18 hours on trypticase soy agar (TSA) plates or in LB broth. Bacteria on plates were fixed by placing a drop of 1% glutaraldehyde onto colonies then a Formvar-coated copper grid was floated on top for ~ 1 minute. A drop of bacteria grown in suspension was placed on a section of parafilm and a Formvar-coated copper grid was floated on top for ~ 30 seconds. The grid was washed by floating on top of a drop of DI water and then fixed with 1% glutaraldehyde ~ 1 minute. Grids from both bacteria grown on plates and in suspension were washed in DI water and stained with 0.75% uranyl acetate for 1 minute before visualization on the electron microscope. At least 10 separate images of each strain grown in each condition where taken and a representative image is shown.

To visualize movement of the *B*. *dolosa* Δ*fliC* mutant, agar concentrations of 0.3%, 0.4%, and 0.5% and differing temperatures of 23°C, 30°C, 37°C, and 42°C were used to grow B. *dolosa* wild-type and Δ*fliC* strains. Wet mounts were prepared and visualized using bright field microscopy.

### Internalization assays

Mid-log phase cultures of *B*. *dolosa* AU0158 wild-type, AU0158 Δ*fliC*, and AU1058 Δ*lafA* strains were mixed with RAW264.7 murine macrophages in 96-well plates at a multiplicity of infection of ~100 bacteria:eukaryotic cell. As a negative control, medium alone was added to eukaryotic cells. The plate was centrifuged at 800 x g for 4 minutes to ensure contact between the bacteria and the eukaryotic cells. After a 2 hour incubation, the DMEM medium was removed and the cells washed with 1X PBS. DMEM containing an antibiotic cocktail [final concentrations: 1 mg/ml kanamycin, 1 mg/ml ceftazidime, 1X Pen-Strep (Sigma Aldrich)] was added to all cells to kill extracellular bacteria. After an additional 2 hour incubation, eukaryotic cells were lysed with 1% Triton X-100 in DMEM, serially diluted and plated on LB agar. Bacterial counts were given in CFU/ml and compared for significance by one-way ANOVA analysis using the Tukey multiple comparison post-test.

### Biofilm assays

*B*. *dolosa* strain AU0158 wild-type, Δ*fliC*, and Δ*lafA* mutants were grown overnight in LB broth, then diluted 1:100 into fresh medium. One hundred microliters of each culture was pipetted into replicate wells of a 96-well PVC plate and incubated for 3 days at 37°C. Biofilm formation was quantitated as described in [59] except: 1X TSB with 1% (w/v) glucose was used for the medium in the wells; biofilms were quantitated after 3 days; and plates were incubated at 37°C in a humidifying chamber (plates were stored on wet paper towels inside a closed container to prevent drying). Biofilm formation was compared between each strain and tested for significance by one-way ANOVA analysis using the Tukey multiple comparison post-test.

### Murine models

All mouse work was performed according to protocol 02791 approved by the Harvard Medical School Institutional Animal Care and Use Committee which is accredited by the Association for the Assessment and Accreditation of Laboratory Animal Care, International (AAALAC). Bacterial inocula of *B*. *dolosa* AU0158 wild-type, *B*. *dolosa* AU0158 Δ*lafA*, and *P*. *aeruginosa* PAO1 wild-type (which served as a well-studied positive control for cytokine production and bacterial persistence) were prepared from dilutions of mid-log phase cultures grown in LB to obtain a solution of ~2.5 x 10^8^ CFU/ml. Six to seven week-old C57BL/6 female mice (Charles River) were anesthetized by intraperitoneal injection of ketamine/xylazine and 10 μl of bacterial inoculum was instilled into each nare (i.e. 20 μL final volume resulting in an inoculum of 5 x 10^6^ CFU/mouse in sterile PBS) as described previously [50]. PBS alone was also instilled in 4 mice as negative controls. Mice were observed twice daily until day 6 as part of routine care.

At 1, 6, 24 and 144 h post-infection, 4–5 mice inoculated with each strain were sacrificed using CO_2_ asphyxiation followed by cervical dislocation, and the lungs harvested and weighed. A bronchoalveolar lavage (BAL) was performed with 1 mL cold PBS and lungs were then homogenized in 1 ml sterile PBS. BAL fluid was separated for immediate serial dilutions for bacterial counts on LB agar plates and then centrifuged. The supernatant was frozen for subsequent cytokine measurement. One-way ANOVAs using the Tukey multiple comparison post-test were used to assess for significance differences between all treatments at each time point.

### Cytokine assays

Measurements of cytokines were performed on supernatants of centrifuged BAL fluid from each mouse by Luminex magnetic assay with a mouse cytokine 15-plex panel (Life Technologies, Grand Island, NY). The following murine cytokines were evaluated: IL-1α, IL-1β, IL-2, IL-4, IL-5, IL-6, IL-10, IL-12 p40, IL-12 p70, IL-17, IL-33, TNF, IFNγ, MCP-1 and GM-CSF. Of these, MCP-1 and GM-CSF failed to produce detectable levels of cytokines in at least 3 biological replicates for either our positive control or test strains and thus were eliminated from analyses.

For *in vitro* ELISAs with cultured murine cells, RAW264.7 macrophage cells were seeded in a 96 well plate and incubated for ~2 hours. *B*. *dolosa* AU0158 wild-type and *lafA* mutant strains and *P*. *aeruginosa* PAO1 wild-type (which served as a positive control) were grown overnight in LB medium, diluted 1:10 in fresh medium, harvested at mid-log phase growth, washed PBS, and resuspended in warmed DMEM medium to the desired concentration. Quadruplicate samples of each bacterial strain or medium alone (which served as a negative control) were added at designated M.O.I.s and incubated for six hours at 37°C and 5% CO_2_. At 6 hours post-infection (h.p.i.), cells were lysed and TNF and MIP-2 were assessed by the Ready-Set-Go! TNF alpha ELISA kit [eBioscience; limits of detection 8 pg/mL] and Mouse MIP-2/CXCL2 ELISA Kit [Boster, limit of detection = 15.6 pg/mL], respectively, according to the manufacturer’s instructions.

For ELISAs on human PBMCs, the buffy coat was collected from an Lymphocyte Separation Medium (Fisher Scientific, Fair Lawn, NJ) gradient, washed in PBS (10 mM, pH 7.2; Gibco, Grand Island, NY) and the PBMCs were plated in RPMI-1640 (Gibco) containing 10% FBS (Atlanta Biologicals, Norcross, GA). PBMCs were incubated overnight at 37°C in 5% CO_2_. They were stimulated the following day with either live or heat-killed (30 min, 65°C) bacteria at an M.O.I. of 10 bacteria:1 PBMC for 20 hours. IL-1β, IL-8, and TNF released into 20 hr cell-free supernatants were measured using eBioscience ELISA Ready-SET-Go! kits (San Diego, CA), according to the manufacturer’s instructions. For all ELISAs, the positive and negative controls performed as expected.

For all cytokine analyses, t-tests were used to compare wild-type and mutant strain induction of cytokines in host cells.

### Transposon library screening

The plasmid pSAM_DTc was created by replacing the erthryomycin cassette from pSAM_DYH [60] with a tetracycline resistance cassette from pmini-CTX1 [61] inserted as a *Mfe*I/*Xba*I fragment. The resulting plasmid, pSAM_DTc was verified by PCR and sequencing and subsequently transformed into *E*. *coli* SM10λpir to generate a donor strain for conjugation.

A transposon mutant library of *B*. *dolosa* AU0158 was created using the pSAM_DTc plasmid and ~200,000 individual transconjugants were collected and grown in LB broth overnight. Twenty colonies from the library were screened by semi-random PCR to ensure random insertion. The library was grown in 25 mL LB overnight and aliquoted into 1 ml freezer stocks containing 20% glycerol. One freezer stock was inoculated into 25 mL LB + 75 μg/ml tetracycline and grown overnight. One mL of this culture was diluted into 25 mL, grown overnight and was then subjected to genomic DNA purification (input). The remaining cells were washed twice with sterile 0.9% NaCl, concentrated to 4.1 X 10^10^ CFU/ml, and 10 μL of this solution was instilled into each nare of 5 C57Bl/6 female mice (6–8 weeks old; Charles River) that had been anesthetized with ketamine and xylazine resulting in the instillation of 8.2 X 10^8^ CFU/mouse. After 48 hours, all mice looked healthy and the mouse lungs were harvested following CO_2_ euthanasia, weighed, homogenized, and inoculated into 25 mL LB broth. Cells were grown overnight in LB liquid, plated on plain LB agar to ensure only the expected colony morphology (i.e. no contamination occurred from gut microbiota), and 1 mL of this was subjected to genomic DNA purification as in [56].

Tn-seq libraries were prepared from output and input libraries according to published procedures [60, 62–64]. Briefly, 50 μg of genomic DNA was restricted using 50U *Mme*I (New England Biolabs) overnight at 37 degrees followed by gel extraction (Qiagen Gel Extraction Kit) to excise a band 1.7–2.1 kb in size. These products were ligated to double-stranded barcoded DNA adaptors using T4 DNA ligase (New England Biolabs) and purified (Qiaquick PCR Purification Kit). Ligation products were used a templates in replicate PCR reactions with high-fidelity polymerase (KAPA Biosystems) and the LIB_PCR_5 and LIB_PCR_3 primers [65]. Pooled replicate reactions were sequenced by Illumina HiSeq 2500 at the Harvard Biopolymers Sequencing Facility. Reads were separated by barcode, trimmed to remove adaptor and transposon sequences, and mapped to the *B*. *dolosa* AU0158 genome using the CLC Genomics Workbench RNA-seq module and default settings as in [60, 62, 63, 66]. The fold change between normalized input and output sample RPKMs for each annotated gene was calculated using the Set Up Experiment function of CLC Genomics Workbench. Statistical analysis of the Tn-seq data was done using the On Proportions function of CLC and the Baggerley’s post-test was used to generate adjusted q-values for each gene. Heat maps representing the input/output fold change for flagella-associated genes generated by MeV software (http://www.tm4.org/mev.html).

A similar protocol was employed to identify genes essential for survival in an intraperitoneal model of infection. The *B*. *dolosa* transposon mutant library was grown overnight in 25 mL of TSB + 75 μg/ml tetracycline, then washed twice in sterile PBS. Two aliquots of 1 mL of this culture were used in genomic DNA extractions for the input samples. Four female 6–8 week old FVB mice were inoculated intraperitoneally with 4.2 x 10^8^ CFU/mouse. After 48 hours, all mice appeared healthy and a peritoneal lavage, the heart, and the spleen were collected from each mouse. Samples were homogenized if necessary (for tissues) and used to inoculate 25 mL LB medium + 75 μg/ml tetracycline. After growth overnight, 1 ml of culture was used for genomic preparation, library prep, and sequencing as described above. The output samples from the spleen did not produce the threshold number of mapped reads (>1 million) and were not included in the analyses.

### Statistics

Unless otherwise stated, statistical analyses were conducted in GraphPad Prism 5.1 using either one-way ANOVAs or t-tests as indicated.

## Results

### Identification and conservation of the *lafA* gene

**Fig 1:**
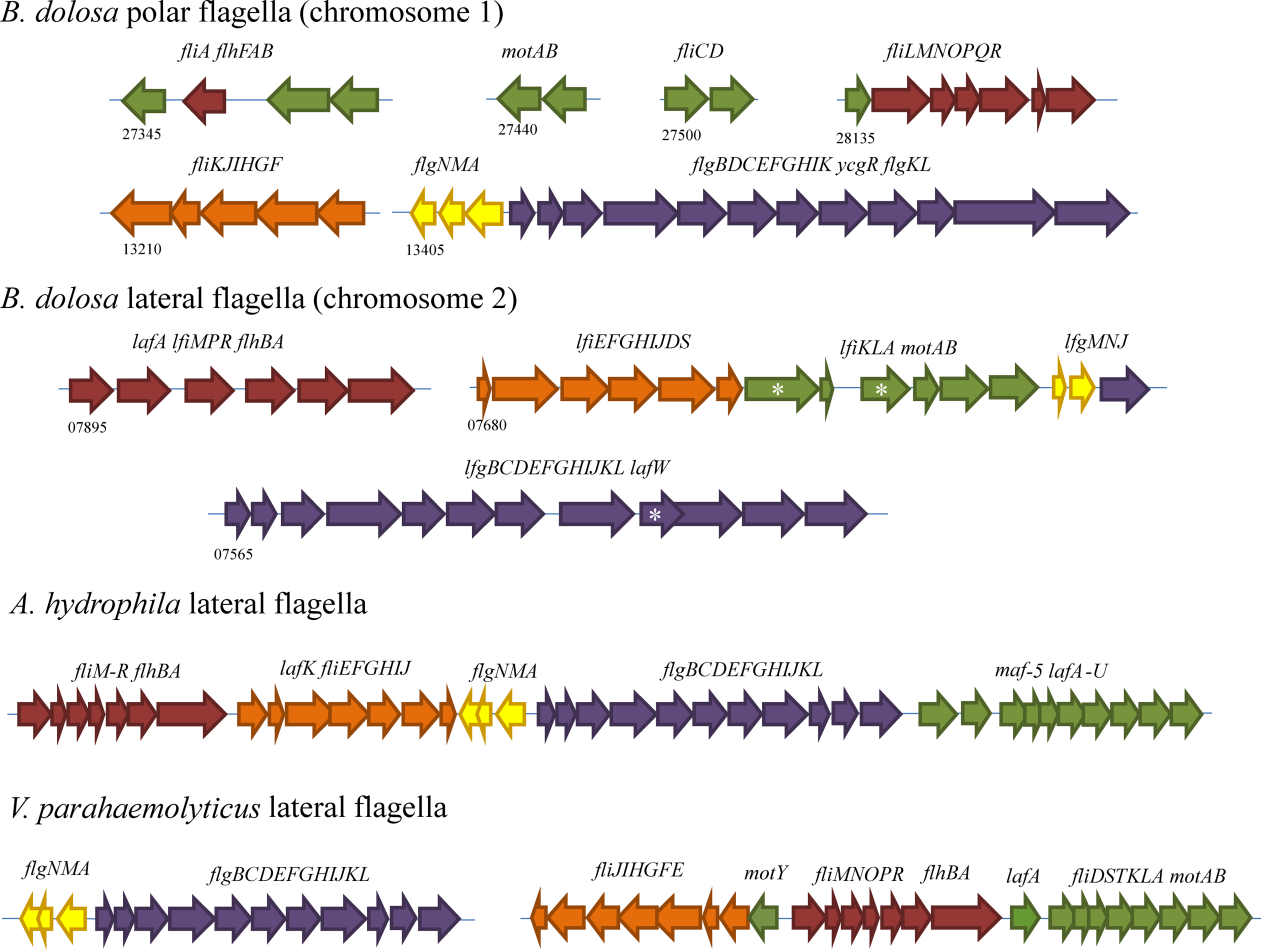
Genomic loci of flagellin in *B*. *dolosa* AU0158, *A*. *hydrophila*, and *V*. *parahemolyticus*. A representation of the placement and conservation of *B*. *dolosa* genes involved in polar flagellin synthesis (above) and the proposed lateral flagellin biosynthesis genes (middle) are shown. For comparison, the well-studied genomic loci encoding lateral flagella proteins from *A*. *hydrophila* [45, 69] and *V*. *parahaemolyticus* [46] are shown. Gene names are given above the groupings and polar flagella genes begin with “fl” while lateral flagella genes begin with “lf” or “laf”. The first *B*. *dolosa* gene in each region has its AK34_RS designation shown. For example, the first gene in the lateral flagella gene set is shown as 27345 for AK34_RS27345. Conservation was determined dynamically using polar flagella homologs as thresholds for e-values. Groups of genes are color coded based on the *A*. *hydrophila* clusters. Asterisks indicate currently unannotated genes in the published *B*. *dolosa* genome sequence.

There are currently two genes in the *B*. *dolosa* AU0158 genome annotated as flagellin. One of these (encoded at the AK34_RS27500 locus) encodes the flagellin for the polar flagellum that has orthology with other polar flagellin genes in and outside the Bcc cluster. The amino acid sequence corresponding to the gene encoding the second flagellin protein, found at locus AK34_RS07895, bears resemblance to the lateral flagellin in other species and we have designated this gene *lafA* for lateral flagellin gene A, consistent with field nomenclature. This gene is bordered on one side by a gene encoding a CheY-like response regulator and on the other by other lateral flagella-associated genes. A further inspection of the genome revealed a number of other flagella-associated genes located in three clusters elsewhere in the genome that are similar to genes encoding lateral flagella biosynthetic proteins in other bacteria species (Fig 1 and S1 Table) and share a low similarity with genes encoding the traditional and more common polar flagella. The organization of the genomic loci in *B*. *dolosa* is distinct from that of *A*. *hydrophila* and *V*. *parahaemolyticus* though there are some similarities. For instance, the *flgBDEFGHIJKL* genes are common located together in polar and lateral flagella systems (Fig 1); however, the location of the *B*. *dolosa lafA* gene is found next to the *fliM* gene rather than next to the *lafB* gene as in *A*. *hydrophila* or the *fliD* gene (a homolog of *lafB*) in *V*. *parahaemolyticus*. The *B*. *dolosa* lateral flagella system also seems to lack lateral flagella-specific genes encoding FliL, FliO, FlhF, FlhG, FliT, FlhC, and FlhD (S1 Table) based on the current annotation and BLAST searches of the nucleotides. FliL is required for flagella rotation [67] while FliO is not required for flagellar biosynthesis [68]. The FlhC, FlhD, FlhF, and FlhG proteins are all transcription factors. It is possible that the polar flagella homologs to these proteins can work with both the lateral and polar flagella systems to lead to motility. Alternatively, the proteins involved in any of the three Type 3 Secretion Systems might also serve roles in the *B*. *dolosa* lateral flagellin system.

**Fig 2:**
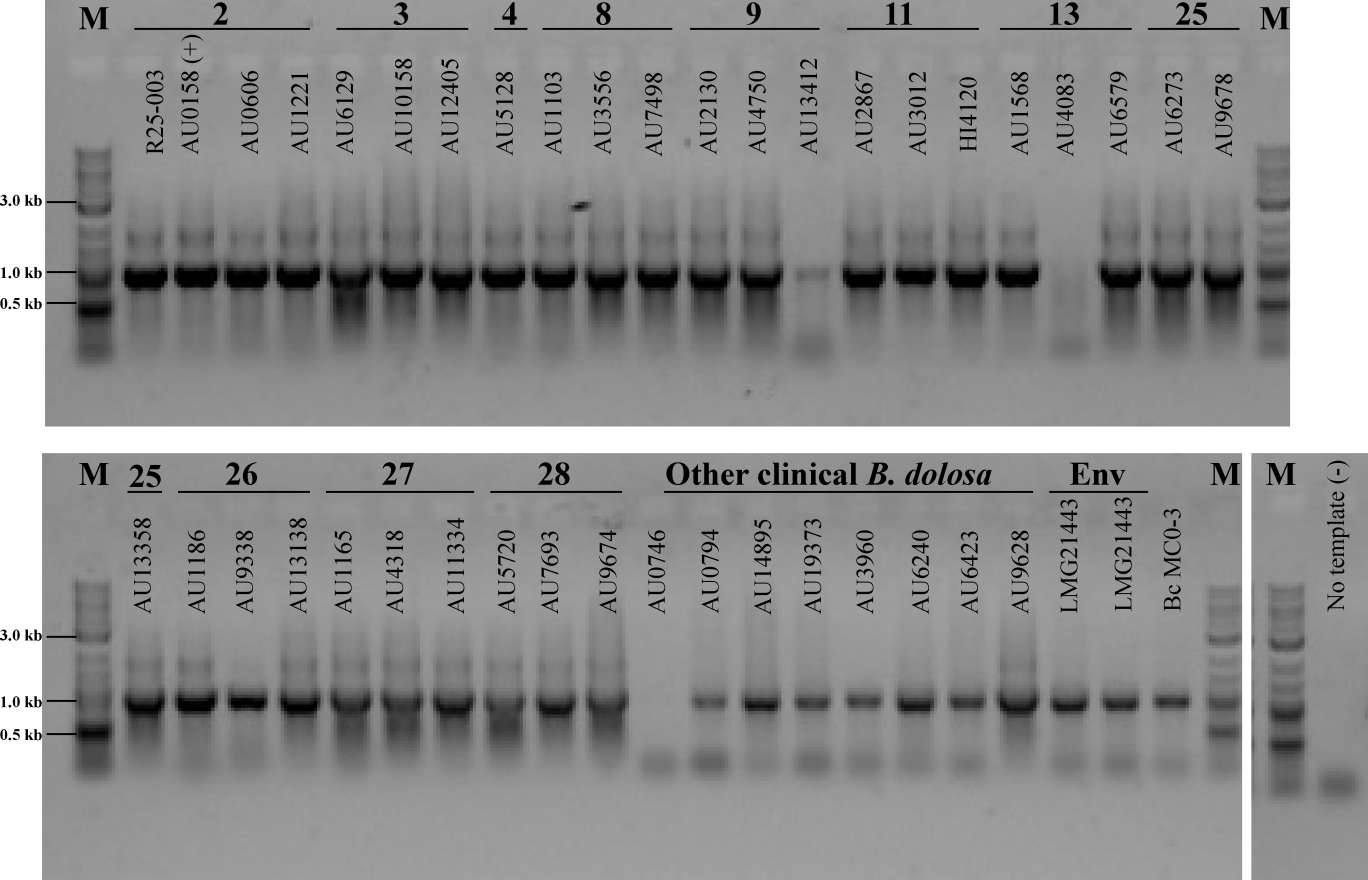
Presence of the *lafA* gene in outbreak and non-outbreak *B*. *dolosa* strains. PCR was performed with primers specific to the *lafA* gene (AK34_RS07895) and the resultant products run on a single 1% agarose gel. Multiple isolates from outbreak patients (patient number on top of lane) or single isolates (for patient 4) from 11 separate *B*. *dolosa* outbreak patients were tested. Also included: other clinical, non-outbreak strains; the sole environmental strain LMG21443 (2 different genome preparations); *B*. *cenocepacia* MC0-3 which contains a *lafA* gene; and a no template negative control. The marker (M) is a 2-log ladder with sizes noted. Gel shown represents three independent replicate PCR reactions.

To determine the conservation of presence of the *lafA* gene in the outbreak strains and other *B*. *dolosa* strains, genomic DNA was prepared from 32 outbreak *B*. *dolosa* isolates from 11 patients (some patients were sampled multiple times) [70] and 9 additional non-outbreak *B*. *dolosa* isolates and used as a template for PCR with primers specific for the *lafA* gene (Table 1). In parallel, we also prepared and used genomic DNA from *B*. *cenocepacia* strain MC0-3 which has a homologous *lafA* gene based on the genome sequence (even though similar homologs are not found in the other sequenced *B*. *cenocepacia* strains). The *lafA* gene was found in almost all outbreak *B*. *dolosa* isolates (Fig 2). It appears to be divergent/absent in 2 outbreak isolates (AU13412 and AU4083) which were obtained from two different patients at two different times during the outbreak. AU4083 was isolated from a patient who later produced an isolate containing the gene which may suggest that there is heterogeneity in the chronic population. The *lafA* gene is also found in most non-outbreak clinical isolates and interestingly, this gene is also found in the sole environmental *B*. *dolosa* strain LMG 21443 which was isolated from the rhizosphere of a maize plant in Senegal. These results together suggest that this gene is found frequently in the *B*. *dolosa* species and is not just a feature of the outbreak strain.

### Amino acid conservation of LafA

**Fig 3:**
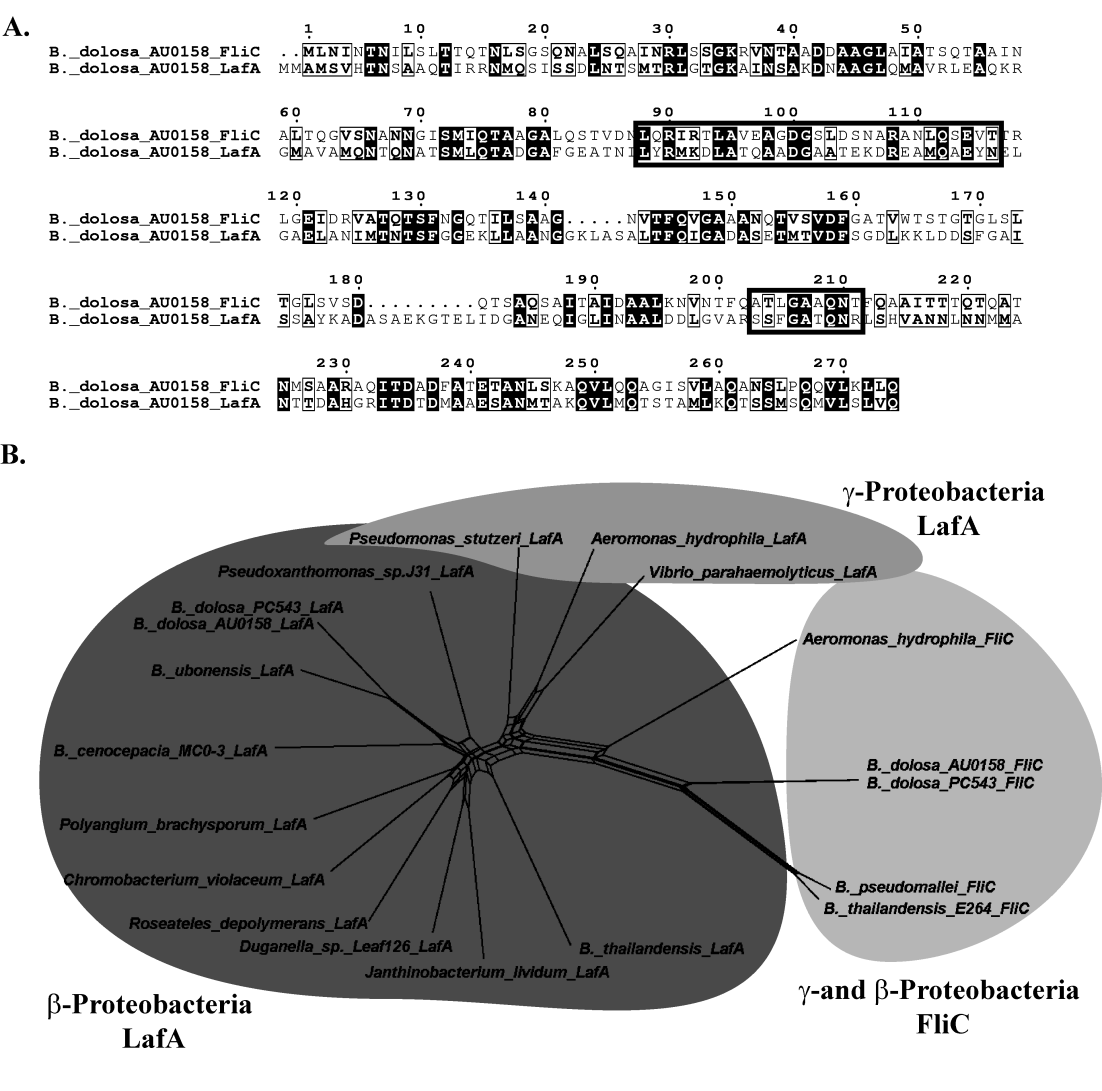
Similarity and conservation of *B*. *dolosa* flagellin proteins. (A) Amino acid alignment between the proteins encoded by the *B*. *dolosa fliC* (AK34_RS27500) and *lafA* (AK34_RS07895) genes shows only weak similarity. Residues in black are conserved and those in lightly outlined boxes are similar. Dark lined boxes indicate the residues predicted to bind to the host immune receptor, TLR5 [71–75]. The alignment of additional homologs can be found in S1 Fig. (B) Phylogenetic network relationship between LafA protein homologs. Three gene clusters are represented by gray shaded areas: the β-Proteobacterial LafA protein sequences including those found in *Burkholderia* species (dark gray on left); a distinct cluster of γ-Proteobacteria LafA proteins (lighter gray on top); and the outgroup of polar flagellins from both β-Proteobacteria and γ-Proteobacteria (light gray on right).

To further demonstrate that the LafA protein encoded at the AK34_RS07895 locus was distinct from the polar flagellin, FliC, we analyzed the protein sequence by comparing it to others in the GenBank and burkholderia.com databases. First, the amino acid sequences for *B*. *dolosa* AU0158 FliC and LafA were aligned and they appear distinct at many residues through there are some similarities, including sites known to be important for TLR5-dependent host immune recognition in other species [71–75] (Fig 3). Additional lateral flagellin proteins were identified in other species and compared to the sequence of the predicted LafA protein using a multi-species alignment (S1 Fig). The *B*. *dolosa* AU0158 LafA protein shows high similarity to a protein in another sequenced *B*. *dolosa* isolate PC543 and a homolog in the related Bcc species *Burkholderia ubonensis*. LafA homologs are also observed in: one *Burkholderia cenocepacia* strain MC0-3 which was isolated from a maize rhizosphere in Michigan; *B*. *cepacia* isolates; *B*. *territorii* which has been found in waters [76]; and *B*. *diffusa* which has been found in both human and natural environments [77]. In addition, proteins homologous to *B*. *dolosa* LafA are found in a number of other β-proteobacterial species including *Burkholderia thailandensis*, *Polyangium brachyosporum*, *Chromobacterium violaceum*, *Pseudoxanthomonas* sp., *Duganella* sp., *Janthinobacterium lividium*, and *Roseateles depolymerans*. It also has a limited amino acid similarity to lateral flagellins in the γ-proteobacteria species *Pseudomonas stutzeri*, *Vibrio parahemolyticus*, and *Aeromonas hydrophila* (S1 Fig). The *B*. *dolosa* LafA proteins are distinct from other LafA-containing strains with at least 16 amino acids that are unique to *B*. *dolosa* (S1 Fig).

We used this protein alignment to assess the phylogeny of this protein using a phylogenetic network. The inclusion of FliC, the polar flagellin monomer, from *B*. *dolosa* AU0158 and PC543, *Burkholderia pseudomallei* and *thailandensis*, and *A*. *hydrophila* served as outgroups. The LafA protein from *B*. *dolosa* was most similar to LafA proteins in other *Burkholderia* strains followed by the rest of the β-proteobacteria (Fig 3). The LafA proteins from γ-proteobacteria clustered separately as did the polar flagella FliC proteins. This suggests that the LafA from *B*. *dolosa* is, in fact, distinct from the polar flagella and may play a role in non-host environments since it is also found in related, environmental, non-pathogenic species.

To further analyze the lateral flagellin conservation, a whole genome alignment was analyzed through the Integrated Microbial Genomes VISTA software. *B*. *dolosa* appears to contain a large region not found anywhere in most other *Burkholderia* genomes (S2 Fig). This region includes three of the lateral flagellin loci as well as genes encoding hypothetical proteins and parts of a Type III Secretion System similar to those found in mammalian pathogens such as *Salmonella*. There is little but some homology of fragments from the *B*. *dolosa* AU0158 genome in this region with other sequenced *Burkholderia* species, especially *B*. *cenocepacia* MC0-3 which has the greatest conservation with *B*. *dolosa* AU0158 of all the species examined. One of the regions surrounding the *lafA* locus is orthologous to regions located on chromosome 2 in other Bcc species while the other border region is more similar to regions found on chromosome 1 in those genomes suggesting that *B*. *dolosa* may have obtained this island from another source and integrated it during a genomic rearrangement event. The GC content of these unique region/island is consistent (64% to 67%) with the genome overall (67%—[11]).

### The effect of mutations in the lateral flagellin gene *in vitro*

To further assess the function of the protein encoded by the *lafA* gene, markerless in-frame mutations were created in AK34_RS27500 (*fliC*; [50]) and AK34_RS07895 (*lafA*). In addition, a *lafA* complemented strain was also created. We tested the effect of these mutations in four agar concentrations representing a gradient of swimming and swarming conditions. The *B*. *dolosa fliC* mutant did not swim past the diameter of the original spotted culture in all agar concentrations and thus was completely non-motile under these conditions (Table 2). The *lafA* mutant, however, swam significantly farther than the wild-type strain in all agar conditions suggesting that the presence of LafA inhibits swimming motility by the polar flagella. The *lafA* complemented strain was not significantly different than the wild-type strain under any condition and was significantly lower than the uncomplemented *lafA* mutant suggesting that the swimming phenotypes and all subsequent phenotypes can be attributed solely to the loss of the *lafA* gene. At the highest concentration of agar, the motility defects of the Δ*fliC* strain were not significantly different than the wild-type but the Δ*lafA* uncomplemented strain still showed a higher degree of motility than wild-type.

**Table 2:** Swimming and swarming motility of flagellin mutant strains.

Strain	0.2% agar^§^	0.3% agar	0.4% agar	0.5% agar
*B*. *dolosa* AU0158 + pUCP18T-miniTn7-Tp	100 ± 12	100 ± 10	100 ± 13	100 ± 23
*B*. *dolosa* Δ*fliC* + pUCP18T-miniTn7-Tp	26 ± 5***	40 ± 7***	44 ± 31***	90 ± 7
*B*. *dolosa* Δ*lafA*+ pUCP18T-miniTn7-Tp	120 ± 13***	136 ± 8***	130 ± 20***	137 ± 22*
*B*. *dolosa* Δ*lafA*+ pUCP18T-miniTn7-Tp-*lafA*	112 ± 12^^^	116 ± 16^^^	103 ± 11^^^	114 ± 7

^§^ Values expressed as a percent of average *B*. *dolosa* AU0158 wild-type diameters and are based on 4–27 biological and technical replicates.

* *p* <0.05

*** *p* < 0.001, based on one-way ANOVAs with Tukey’s multiple testing correction. compared to *B*. *dolosa* AU0158 wild type. Two-way ANOVA analysis indicates a significant interaction (*p* = 0.0051) between strain (p < 0.0001) and agar concentration (*p* = 0.0042).

^^^ Indicates significant differences between *B*. *dolosa* Δ*lafA*+ pUCP18T-miniTn7-Tp and *B*. *dolosa* Δ*lafA*+ pUCP18T-miniTn7-Tp-*lafA* complemented strains based on one-way ANOVAs with Tukey’s multiple testing correction.

The contribution of LafA to host cell internalization was analyzed by infecting RAW264.7 murine macrophages with wild-type *B*. *dolosa* AU0158 or the *B*. *dolosa lafA* deletion mutant. There were no significant differences between the wild-type and mutant strain in four replicate experiments (S3 Fig). Likewise, there were no significant differences in biofilm formation between the wild-type and *lafA* mutant strains based on crystal violet staining (S4 Fig). These data suggest the putative lateral flagella do not play a role in macrophage invasion/phagocytosis or in biofilm formation.

**Fig 4:**
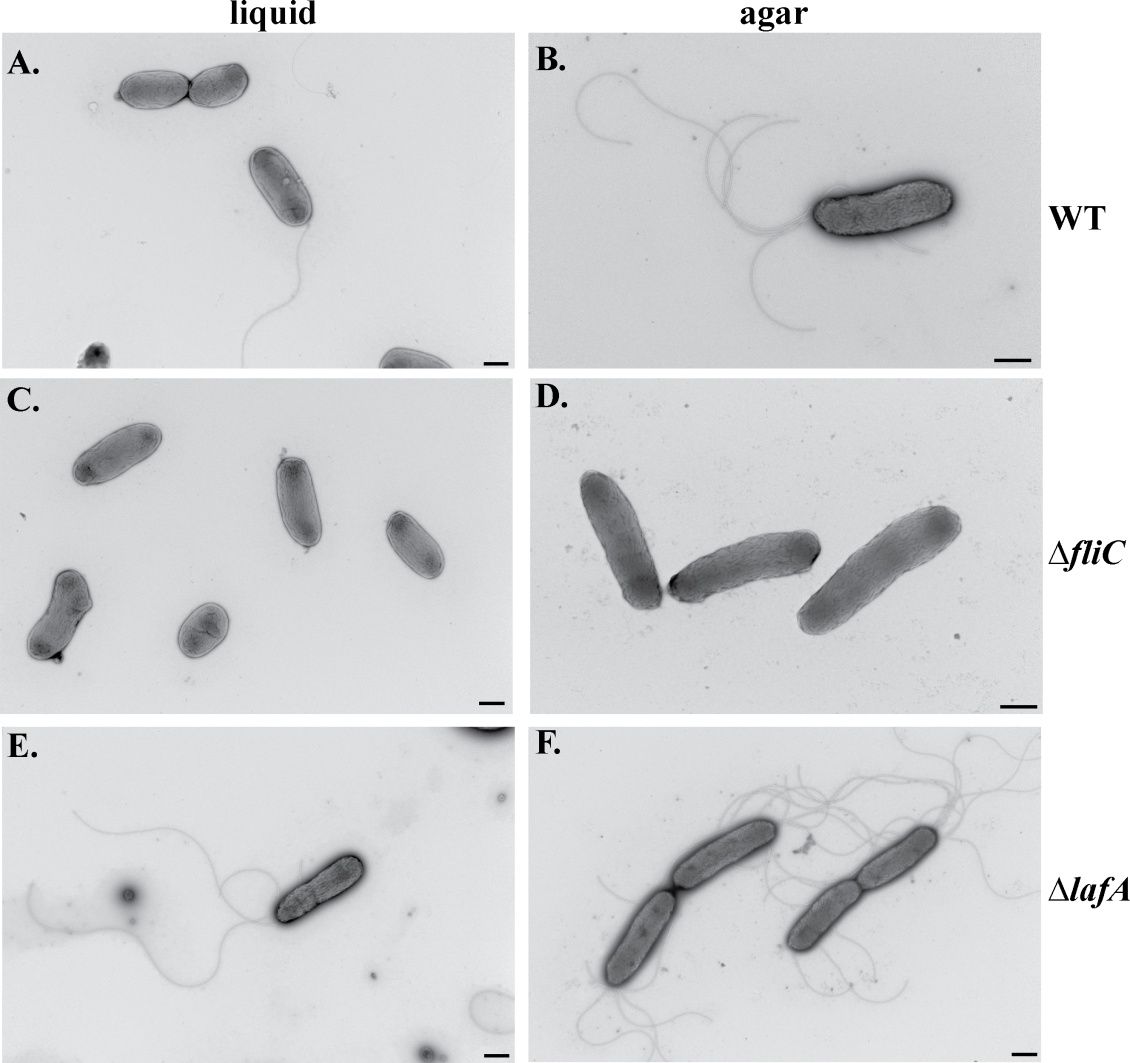
Production of *B*. *dolosa* flagella in nutrient-rich media. *B*. *dolosa* strains were grown in either LB liquid medium (panels A, C, and E) or grown on TSA plates (panels B, D, F) then subjected to transmission electron microscopy. Representative images are shown for *B*. *dolosa* wild-type (panels A and B), *B*. *dolosa* Δ*fliC* (panels C and D), or *B*. *dolosa* Δ*lafA* (panels E and F). Black bars indicate 500 nm scale. Representative images of 10 replicate images are shown.

The production of flagella was also examined using transmission electron microscopy. When grown to stationary phase in a nutrient rich liquid medium, wild-type *B*. *dolosa* has few attached flagella but when observed, there is a single flagellum extending from one end (Fig 4). Because there are a number of flagella observed in the background of these images (S5 Fig), it is likely they were shed upon entering stationary phase. On an agar medium appears to have a few polar flagella extending from a pole in a manner more similar to a lophotrichous arrangement rather than a peritrichous arrangement (Fig 4). The higher number of flagella found on solid surfaces has also been observed in *Burkholderia glumae*, a plant pathogen that lacks a homolog to the *lafA* gene, and the number and localization of the flagella in this organism has been shown to be condition-dependent [78].

The *B*. *dolosa* Δ*fliC* mutant lacks a detectable flagellum in liquid or on solid surfaces (Fig 4). We note here that under low agar conditions (concentrations of 0.3–0.5%) and growth at 30°C, the *B*. *dolosa* Δ*fliC* mutant is able to observably move both on agar plates and using wet mounts under bright field microscopy. Whether this is due to Brownian motion, Type IV pili-mediated twitching motility (for which *B*. *dolosa* AU0158 has the genes), or the function of lateral flagella remains to be determined.

Interestingly, the *B*. *dolosa* Δ*lafA* mutant appears to have a 1–2 flagella when grown in liquid (Fig 4) but shows 2–8 flagella extending from a single pole when grown on an agar medium (Fig 4). This strain, however, should not produce lateral flagellin monomers which suggest that these may be extra polar flagella. Together the data shown in Fig 4 suggest that the Δ*fliC* mutant is not motile due to a lack of lateral and polar flagella under these conditions while the extended swimming phenotype of the Δ*lafA* mutant appears to be due to the overproduction of flagella in these cells. The number of flagella extending from each cell was quantitated from 4–12 images taken in replicate trials (examples shown in S5 Fig) and this data is presented in Table 3.

**Table 3:** Quantitation of the flagella from multiple TEM images.

Strain	Condition	# of cells	Mean	Median	Range	Statistically different than WT?^†^
*B*. *dolosa* WT	stationary/liquid	25	0.16	0	0–2	—
*B*. *dolosa* Δ*fliC*	stationary/liquid	21	0	0	0	No
*B*. *dolosa* Δ*lafA*	stationary/liquid	19	0.11	0	0–1	No
*B*. *dolosa* WT	agar	18	2	2	1–5	—
*B*. *dolosa* Δ*fliC*	agar	45	0	0	0	Yes ***
*B*. *dolosa* Δ*lafA*	agar	21	3.6	4	1–8	Yes ***

^†^ based on one-way ANOVAs using Tukey’s multiple testing correction.

*** *p* <0.001.

### The effect of mutations in the lateral flagellin gene *in vivo*

*B*. *dolosa* AU0158 has the ability to persist in mouse lungs for more than 6 days without causing symptoms even at high doses [38]{Roux, 2017 #7359}. To determine if the lateral flagella plays a role in host association and persistence *in vivo*, the wild-type and lateral flagella deletion mutant strains were inoculated intranasally into 4–5 C57BL/6 mice and monitored over the course of 6 days. At 1, 6, 24, and 144 hours post-infection, bronchoalveolar lavages (BALs) were collected for bacterial survival analyses and cytokine expression and then the lungs were removed and homogenized for bacterial counts. *P*. *aeruginosa* PAO1 served as a positive control because its behavior and cytokine recruitment has been well-described in the published literature. As a negative control, mice inoculated with the PBS vehicle alone were run in parallel.

**Fig 5:**
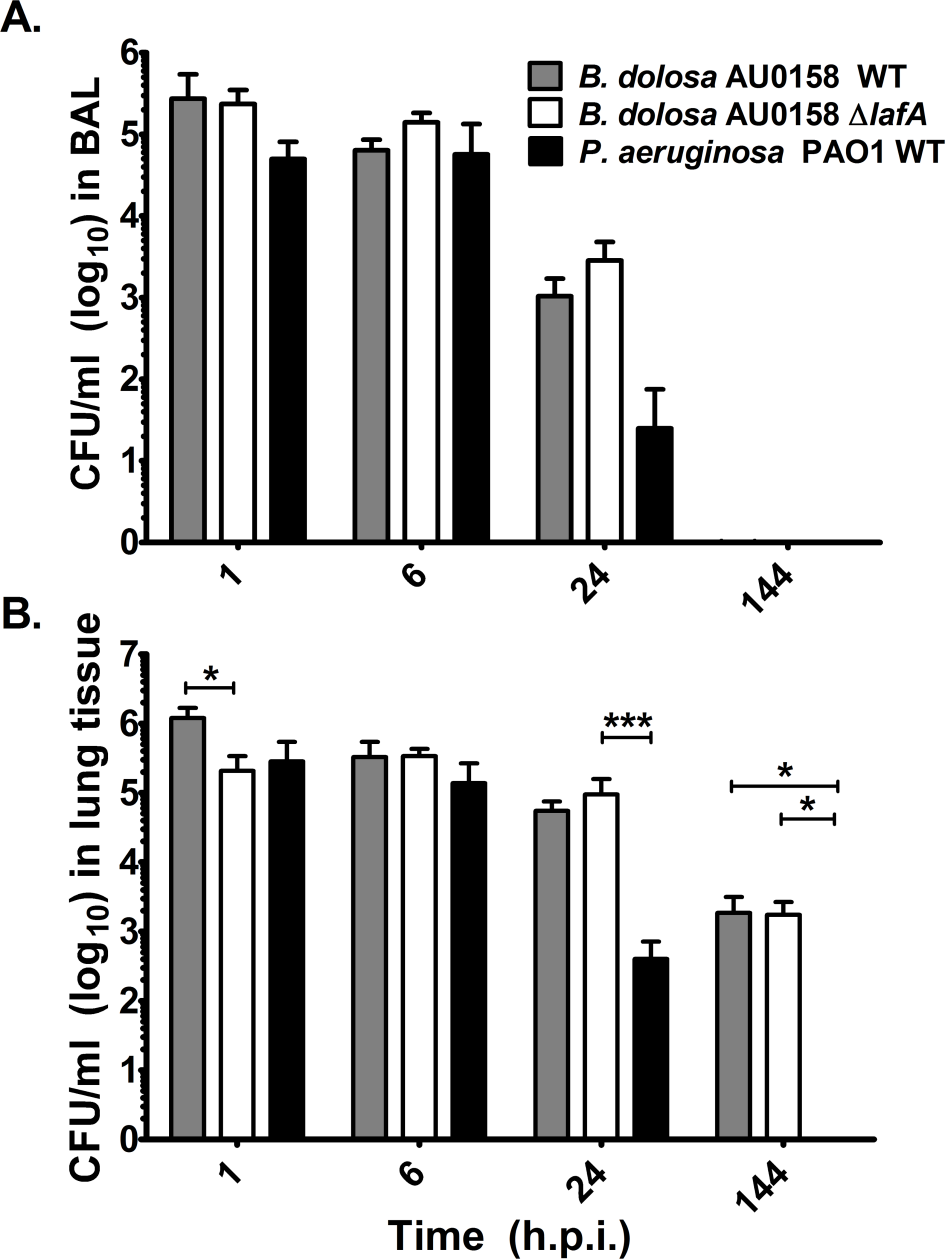
Bacterial survival in murine pneumonia model. C57BL/6 mice were inoculated intranasally with 5 x 10^6^ CFU/mouse and observed over time. At indicated times, the BAL fluid (panel A) was collected and the lungs homogenized (panel B), serially diluted, and plated for bacterial counts. Error bars represent one standard deviation of the data from 4–5 mice/group. Significant differences were assessed using one-way ANOVA. Asterisks indicate strain-based differences assessed using the Tukey’s multiple comparison test. *p < 0.05, ***p < 0.001. There were no significant differences between strains in panel A. The levels of bacterial survival in the wild-type controls was published previously [50] as the bacterial survival analysis of the *B*. *dolosa lafA* deletion mutant was completed as part of a larger study.

**Fig 6:**
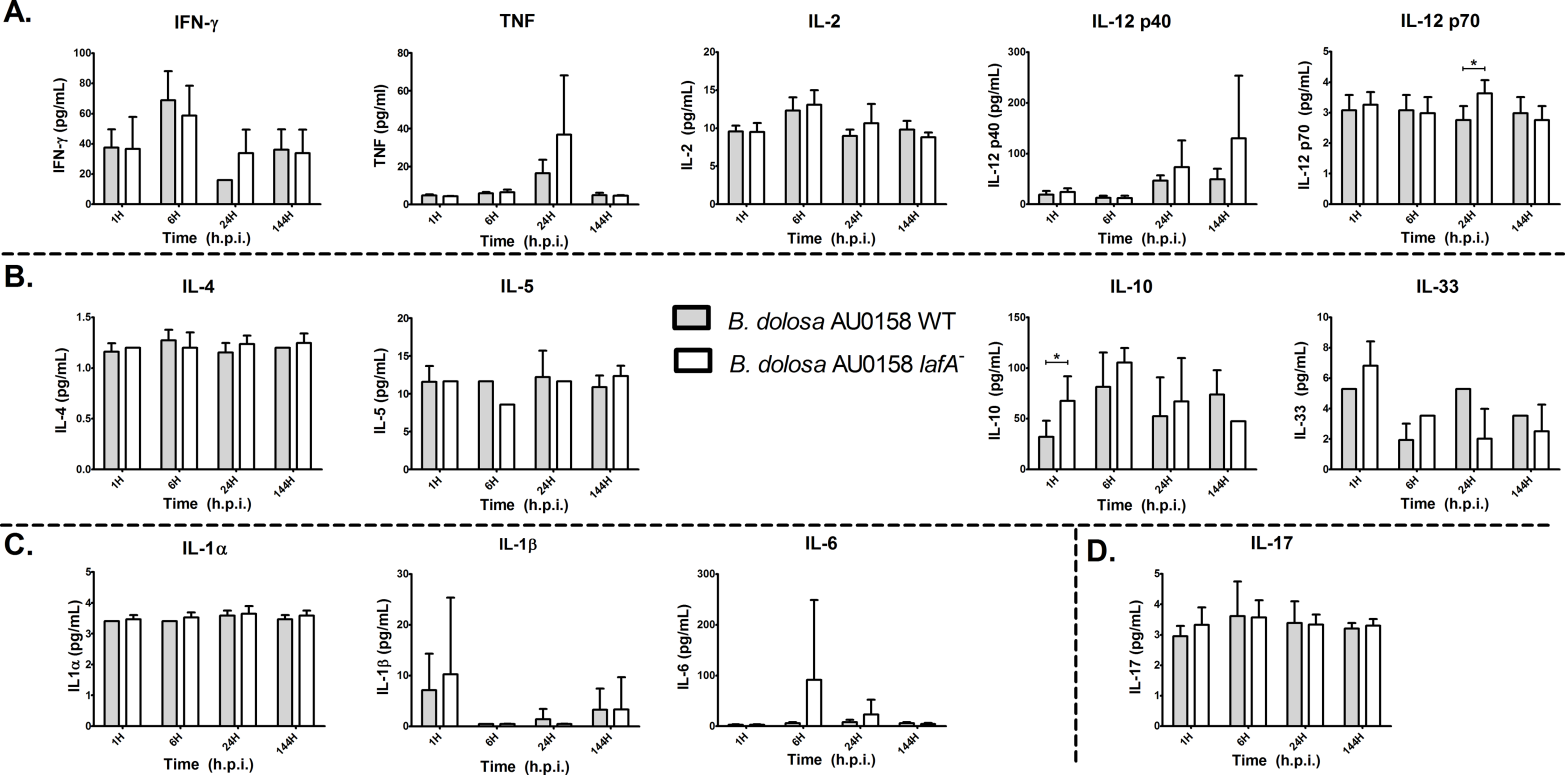
Cytokine expression in the murine lung during infection with *B*. *dolosa* wild-type or *lafA* deletion mutant strains. Cytokines belonging to the Th1 response pathway (panel A), Th2 pathway (panel B), innate/acute immunity (panel C), and the Th17 pathway (panel D) were collected from the BAL fluid from 3–5 mice and measured using Luminex protocols. Gray bars represent *B*. *dolosa* AU0158 wild-type strain and the white bars represent the *B*. *dolosa* AU0158 *lafA* deletion mutant strain. Error bars represent one standard deviation. Significance was assessed by unpaired t-tests between strains at each time point. An asterisk indicates a *p*-value < 0.05.

As expected, the wild-type *B*. *dolosa* strain persisted for up to 24 hours in the BAL and 144 hours in the lung tissue, presumably due to their intracellular nature in host cells (Fig 5). The *lafA* mutant was found at similar levels such that there were no significant differences in bacterial viable counts between the wild-type and *lafA* deletion mutant strain at most time points other than 1 hour after infection, when counts of the *lafA* deletion mutant were about 5-fold lower than the wild-type strain in the lung tissue. Similarly, the expression of 13 different cytokines was not significantly different between wild-type and *lafA* deletion mutant strains in almost all cases (Fig 6) suggesting that *lafA* is not playing a significant role in host response survival *in vivo*.

**Fig 7:**
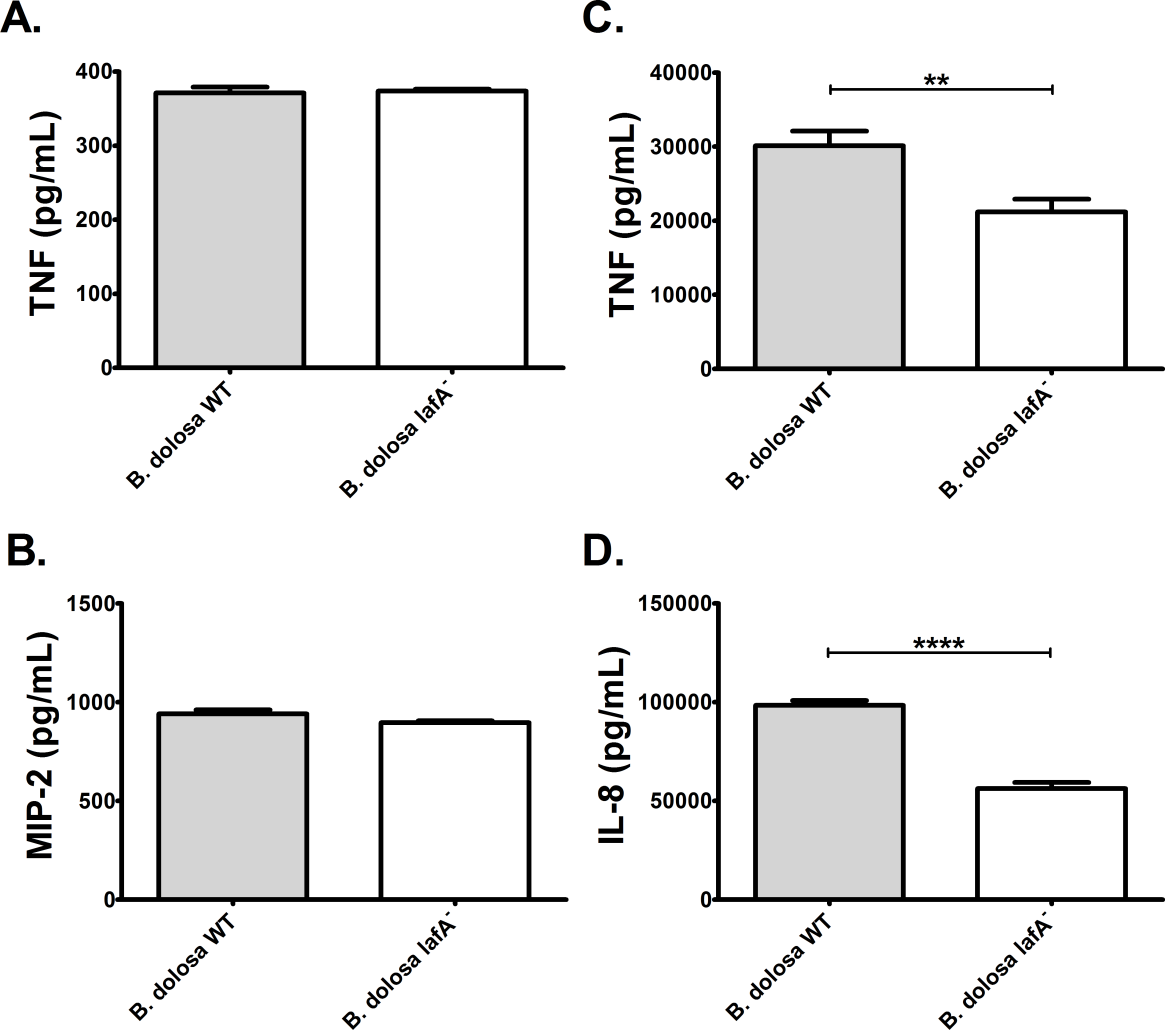
*In vitro* cytokine response from human and mouse cells. *B*. *dolosa* AU0158 wild-type and *lafA* deletion mutant were added at an M.O.I. of 10 to RAW264.7 murine macrophages to (panels A and B) or human PBMCs (panels C and D). The production of TNF or IL-8/MIP-2 was assayed for each strain from replicate samples. Medium alone negative controls were near or below the limit of detection (N.D.) for all panels. Error bars represent one standard deviation of the data. Asterisks correspond to *p*-values of <0.01 (**) or <0.0001 (****) based on unpaired t-tests.

It has been noted in prior studies that wild-type *B*. *dolosa* does not produce robust cytokine expression in the murine lung possibly due to a suppression of the immune response; however, it can produce a pro-inflammatory cytokine responses far above the limit of detection in both human and murine cell lines [50]. To test whether the *B*. *dolosa lafA* deletion mutant is altered in its ability to induce a cytokine response in cultured or primary cells, we stimulated RAW264.7 murine macrophages with *B*. *dolosa* AU0158 wild-type and the *lafA* deletion mutant strains and measure the levels of the pro-inflammatory cytokines TNF and MIP-2 (an IL-8 homolog). The production of cytokines from this cell line was moderate but detectable after bacterial infection and there were no significant differences between the wild-type and mutant strains (Fig 7). This agrees with previous studies in which *B*. *dolosa* produces a moderate cytokine production in these cells that is not significantly different from that produced by a mutant lacking the polar flagellum [50].

We also tested the effect of the *lafA* mutant on eliciting cytokines from human peripheral blood mononuclear cells (PBMCs). Interestingly, the TNF and IL-8 cytokine responses in PBMCs were robust, and significant differences were observed between the wild-type and *lafA* deletion mutant strains (Fig 7), with the *lafA* deletion mutant inducing 1.4-fold less TNF and 1.7-fold less IL-8 production from host cells, suggesting that LafA plays a role in recognition by the host immune response. Taken together with previous data, this suggests that the role of LafA in host immune recognition may be masked *in vivo* by an unknown mechanism as was proposed previously for the polar flagellin [50].

### The essentiality of the lateral flagella *in vivo*

To determine if the lateral flagella was essential for colonization *in vivo*, we created a transposon mutant library of *B*. *dolosa* AU0158 and used this library as an input for mouse lung colonization or septic dissemination via intraperitoneal injection. After 48 hours inside the mouse, the mutant libraries were cultivated from the tissues and the frequency of all transposon mutants was measured by counting the number of transposon-junction fragments in the input and output pools. Those genes with reads that were significantly reduced (fold changes < -2) in the outputs are deemed important under these conditions as mutations in these genes did not survive as well as bacteria containing mutations in other genes. Those mutants found at higher levels in the outputs (fold changes > 2) are usually thought to contain transposon insertions in genes encoding proteins or regulatory RNAs involved in immune recognition; thus mutations in these genes will survive better than the general population as they will be less well recognized by the host.

**Fig 8:**
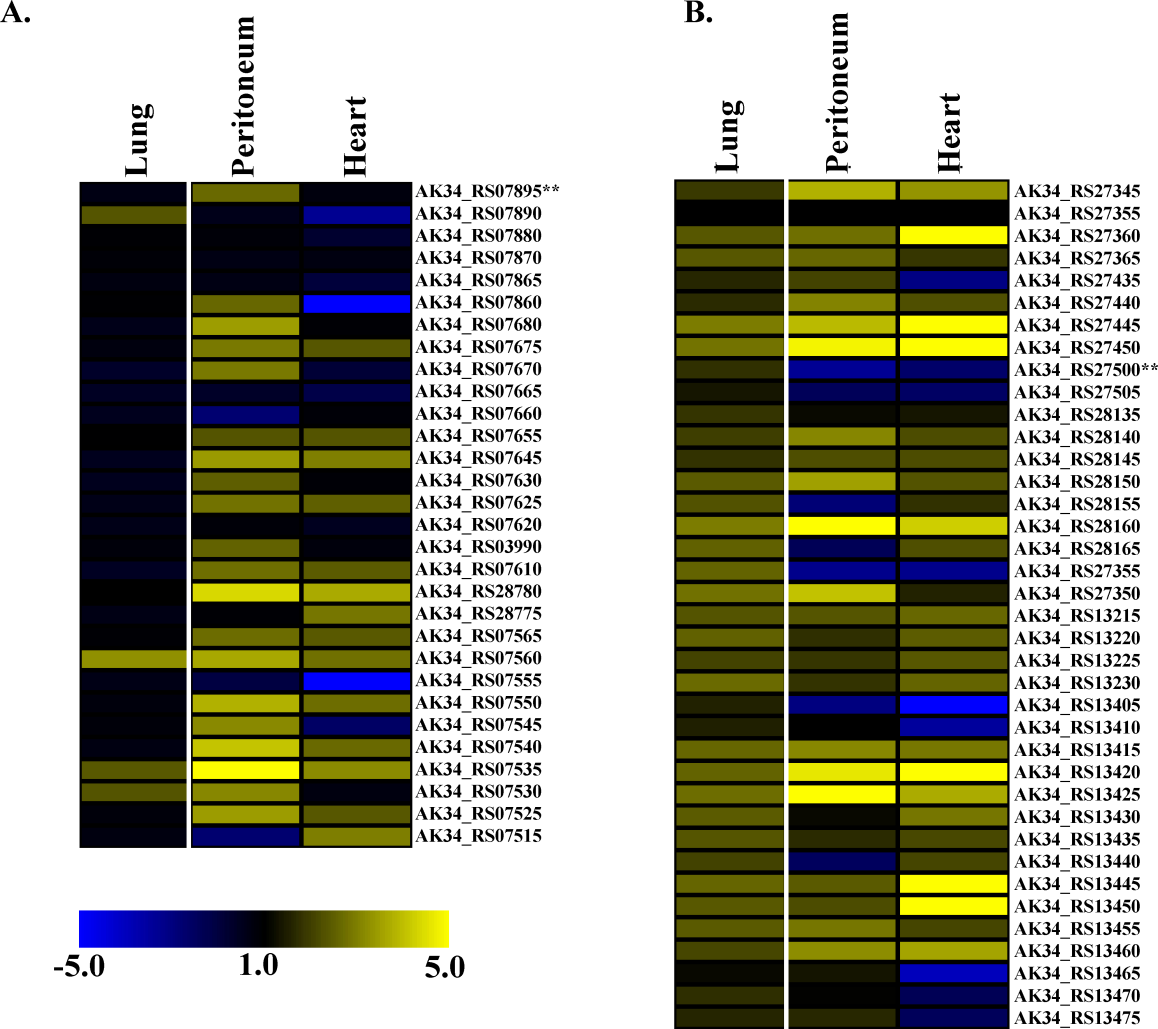
The effect of transposon insertions in the polar and putative lateral flagella genes on lung colonization or septic dissemination of *B*. *dolosa*. Insertion site amplification was performed on *B*. *dolosa* AU0158 transposon libraries from input or after three selections from two different experiments–mouse lung colonization (“Lung”) or peritoneal injection followed by septic dissemination (site of injection–“Peritoneum”, dissemination–“Heart”). Colors indicated the fold change between input and output samples as indicated in the blue-to-yellow key. Results given for genes suspected to be involved in lateral flagella biosynthesis (panel A) or polar flagellin synthesis (panel B).

We observed that most genes involved in lateral flagella synthesis (34 total) were not required for colonization of the mouse lung to a high degree (Fig 8) with most fold changes between -1 and -1.55. For only 11 genes was this decrease significant (*q* values <0.05) suggesting that the lateral flagella may only play a weak or indirect role, if any, in murine lung colonization. One exception to the general downward trend of these mutants is that mutations in AK34_RS07560, which encodes putative lateral flagella homolog to the basal body rod protein (FlgB in polar flagella), that showed a 2.42-fold increase between output and input in the lung and a 2.99-fold increase in the peritoneum, suggesting that interruption of this gene may improve survivability in the murine lung and peritoneum.

In the peritoneal model, two outputs were evaluated: a peritoneal lavage and heart tissue. For almost no gene was there a significant decrease based on adjusted *q* values even though there fold changes between -5.85 and 13.92. This was due to large variability in these data, so it is difficult to make conclusions whether lateral flagella are playing a role in lung colonization or septic dissemination under these conditions.

This is in contrast to the data for the polar flagella system of *B*. *dolosa*. There is a small but significant effect of polar flagella mutations in the murine lung with 24 of the 38 associated genes showing a 2-fold increase or more after colonization, all of which are significant (Fig 8). In the septic dissemination model, there are 20 and 18 genes that show an increase of 2-fold or more in the peritoneum and heart outputs respectively though very few of these are significant. These data are consistent with expectations that polar flagella mutants may fair better in lungs and the blood stream as they will be less recognized by the TLR5 host immune receptor and thus cleared less rapidly. It also suggests that mutations in the gene encoding the polar flagella biosynthetic proteins are fundamentally different than those encoding the genes encoding the lateral flagella biosynthetic components.

We further looked at additional genes that were required for murine lung colonization that were putatively not involved in flagella production. Of the 293 genes that showed a fold change greater than 2 and had *q*-values <0.05, there are a number of genes encoding proteins involved in phenylacetate and anthranilate metabolism, pilus assembly, and sensory or transcriptional signaling systems (S2 Table). Mutations in the gene encoding the LexA transcriptional repressor seemed to be particularly unfit in the lung colonization model as this gene was under-represented 55-fold in output compared to input samples.

Also, the 6 genes that encode the TolQRAB-Pal outer membrane complex (BDAG_02395–2400) are all highly required for mouse lung colonization. In other species, this complex is known to play a role in membrane integrity, antibiotic resistance, and cell division. However, the role of this complex in virulence or in host colonization has not been described to date so whether the requirement for this complex is direct (truly deficient in lung colonization) or indirect (generally sick) remains to be explored.

Genes required for septic dissemination were also examined and we found only 182 genes that showed a 2-fold change and *q*-values of <0.05 between out and input samples (S3 Table). Within this group, there are genes required for peritoneal colonization and septic dissemination into the heart including components of the Flp pilus, transcriptional regulators, and several major facilitator family proteins, which are usually involved in membrane transport of small molecules. Conversely, mutated genes that were over-represented in the outputs seemed to have no patterns in terms of functional groupings or proximity of genetic loci outside of the genes encoding flagella-related proteins. Together, these in vivo results suggest that there are a number of genes required for lung colonization and these genes are different than those required for a septic dissemination/intraperitoneal colonization model. These genes will need to be explored in future studies.

## Discussion

The discovery of the lateral flagellin system in *B*. *dolosa* is surprising in that it has yet to be reported in any other Bcc species, despite the plethora of genomes available for this group. Our analysis also shows that the genetic loci that comprise the presumptive lateral flagella system in *B*. *dolosa* is unique compared to that in the well-characterized pathogen *A*. *hydrophila* in that it is not found in one single chromosomal locus but rather is spread over three regions on the second chromosome (Fig 1). A survey of additional literature also shows that it is distinct from other organisms bearing lateral flagella systems. For example, *Vibrio parahemolyticus* has two regions containing all genetic loci required for lateral flagella (Fig 1), one on the major chromosome and one on the minor chromosome [46, 79], and the order and composition is different from what is observed in *B*. *dolosa*. For example, there does not appear to be a *motY*_*L*_ gene in *B*. *dolosa*. Similarly, differences are observed in the order and composition of lateral flagella genes in other *A*. *hydrophila* and *A*. *caviae* strains [45, 49], and in the pathogenic 042 strain of *E*. *coli*, which also bears a lateral flagella system [80].

The bioinformatic finding that there is a *B*. *dolosa* LafA homolog in the soil-, water-, and plant-associated β-proteobacteria *Duganella*, *Janthinobacterium*, *Pseudoxanthomonas*, *Roseatales*, *Polyangium*, and *Chromobacterium* suggests that the presence of this system is perhaps more ancestral than previously recognized. This observation is also supported by the fact that the *B*. *dolosa* LafA is similar to other *Burkholderia* LafA homologs and distinct from those found in γ-proteobacterial strains (Fig 3) suggesting it was not recently acquired in this species.

The *lafA* gene is notably absent from most Bcc isolates despite the plethora of genomes available for organisms within this complex. This could suggest that perhaps *B*. *dolosa* is more ancestral than other members of the complex which may have lost these genes over time. Since the resolution of the phylogeny of the Bcc is hampered by their high genetic relatedness, the true phylogeny of *B*. *dolosa* cannot be inferred accurately yet; thus, it is difficult to determine if it is less derived than other Bcc species. Conversely, it could be that *B*. *dolosa* acquired these genes from a related, high GC species shortly after diverging from the rest of the Bcc. Homologous genes are found in at least one isolate of *B*. *cenocepacia* (MCO-3) and in only a few other *Burkholderia* species, including *B*. *thailandensis* and *B*. *pseudomallei*, which would support the hypothesis that this gene was lost from many strains over time as it is less likely to be acquired by many phylogenetic lines through horizontal gene transfer.

There have been a number of studies that examine the hierarchical transcriptional regulation of the lateral flagella systems in *A*. *hydrophila*, *V*. *parahemolyticus*, and *E*. *coli* 042. In these species, the σ^54^ and σ^28^ proteins (encoded by the *rpoN* and *fliA* genes), along with the transcriptional regulators FlhD, FlhC, and FleQ, coordinate the expression of the late genes involved in flagella maturation [46, 81]. *B*. *dolosa* has multiple homologs of *rpoN* (AK34_RS14460 and AK34_RS10110), *fliA* (AK34_RS27345 and AK34_RS07625), and *fleQ* (AK34_RS04955 and AK34_RS04950) but only one homolog each that shows similarity to *flhD* and *flhC*. This suggests that perhaps *B*. *dolosa* shares some regulators between the polar and lateral flagella–a phenomenon also observed in *V*. *parahemolyticus* and *Azospirillum brailense* [82, 83] but not in *Aeromonas* species [47, 81]. The change in the production of the polar flagella observed by transmission electron microscopy supports the notion that the regulation of expression for these two flagellar systems is intertwined as a deletion in the *lafA* gene leads to the overproduction of the polar flagella (Fig 4) and an increase in the swimming ability of this strain compared to wild-type (Table 2).

Using transmission electron microscopy, the polar flagella of *B*. *dolosa* is observed in the wild-type and mutant strain lacking the lateral flagellin gene though the localization and/or number of flagella differs in the latter. The lack of observable lateral flagella in the polar flagellin mutant strain is surprising given the putative role for lateral flagella in movement over semi-solid surfaces and could suggest that either the conditions for our assays will not allow mature lateral flagella production, that the lateral flagella of *B*. *dolosa* cannot be stained by uranyl acetate, or the lateral flagella in *B*. *dolosa* are used for other purposes beside swarming, such as movement inside host cells. The overproduction of flagella in the *B*. *dolosa* Δ*lafA* mutant is also somewhat surprising as this strain lacks the gene for the monomer that comprises the putative lateral flagella. This suggests that the additional flagella we observe are either mis-regulated polar flagella or that the FliC flagellin monomer for the polar flagella can be assembled on the lateral flagella machinery; however, the latter option seems less likely given the number of amino acid differences in these two flagellin proteins (Fig 2).

The role of the lateral flagella is thought to be beneficial for human pathogens because it may allow for greater attachment to host cells [48, 49, 84–87], better biofilm formation [48, 49, 88], and thus better colonization of hosts. In most cases, it is assumed that the polar flagella will allow the bacterium to travel greater distances to find new hosts. Once bound, the lateral flagella may facilitate spread of the bacterium on the surface of the host tissues. Our assays showed that the *B*. *dolosa lafA* gene is required neither for biofilm formation (S4 Fig) nor internalization by murine macrophages (S3 Fig). This is inconsistent with the published studies in *Aeromonas* species in which it was shown that the lateral flagella are required for biofilm formation [48, 49]. The reason for this discrepancy is not clear though we speculate that perhaps the long divergence between the γ-proteobacteria *Aeromonas* species and the β-proteobacteria *B*. *dolosa* has also contributed to a divergence in the function of the lateral flagella.

While the function of the *B*. *dolosa* lateral flagella is not known, a study from French *et al*. has shown that the proposed lateral flagella of the closely related *B*. *thailandensis* contributes to intracellular swimming but not swarming on soft agar [89]. In this previous study, time lapse images show the effect of mutation in the motor component of this system and their striking inability to move inside cultured cells in contrast to the wild-type. The LafA protein located at locus BTH_II0151 in *B*. *thailandensis* E264, is ~54% identical to the predicted lateral flagellin proteins located at the AK34_RS07895 locus in *B*. *dolosa* (*lafA*), the Bcenmc03_4651 locus of *B*. *cenocepacia* MC0-3, and has even higher homology to proteins encoded in some, but not all, *B*. *pseudomallei* strains (>96% identity). So, even though the select agent, BSL-3 pathogen *B*. *pseudomallei* has been extensively studied for virulence factors, the lateral flagella have yet to be implicated in human pathogenesis. In our study, a strain lacking the lateral flagellin gene was not defective in murine lung colonization (Fig 5), macrophage internalization (S3 Fig), or septic dissemination (Fig 8) which we speculate could be because the function of this particular lateral flagella system is not for host cell adherence, invasion, or swimming in liquids such as blood by this pathogen.

Very little is known about *B*. *dolosa* specifically, and even less is known about its ability to cause disease or why it caused an outbreak at Boston Children’s Hospital. Our study showed an effect for the *B*. *dolosa* lateral flagellin deletion mutant that was not observed for a *B*. *dolosa* polar flagellin deletion strain—in primary human PBMCs, we observed a modest but significant decrease in the cytokine response to the lateral flagellin deletion mutant compared to the wild-type strain. We suspect this may be due to the ability of host receptors to recognize polar flagellins, which bear some homology to the lateral flagellin. However, it was previously shown that *B*. *dolosa fliC* mutants were not significantly different from wild-type in terms of cytokine production [50], suggesting that they have either evolved a polar flagellin that is incapable of being recognized by the TLR5 or there are other factors that participate in the host sensing of lateral flagella. It should be noted that the use of wild-type mice to simulate cystic fibrosis-like conditions is not ideal even for examining virulence or immune responses in CF pathogens such as *B*. *dolosa*. It was previously shown that macrophages from CF patients are deficient in autophagy, fail to efficiently clear CF pathogens including *Burkholderia cepacia* complex isolates [90–94], and exhibit a heightened cytokine production associated with defects in the CFTR gene [95]. This may contribute to the lack of functional cohesion in the genes required for lung and peritoneal colonization in the Tn-seq screen (S2 and S3 Tables) as many of the genes had no seemingly obvious role in host cell attachment or the stress response as might be expected in a *CFTR* deficient murine model. The basis of future experiments will focus on the ability of *B*. *dolosa* AU0158 strain to invade and replicate inside human *CFTR*-deficient macrophages and mice, to better understand the role of the TolQRAB complex in lung colonization, and the potential production, uses, and regulation of the lateral flagellin system of *B*. *dolosa in vitro* and *in vivo*.

## Supporting information

S1 Fig Amino acid alignment of LafA sequences. Amino acid sequences for lateral flagellins were obtained from GenBank and aligned using ClustalOmega. The resultant alignment was put into ESPript software to aid in visualization. The top three sequences belong to γ-proteobacteria species, the next 11 belong to *Burkholderia* strains and species, and the remaining six sequences belong to other β-proteobacteria species. Note that three Bcc species, *B*. *cepacia*, *B*. *territorii*, and *B*. *diffusa*, have two homologs of the LafA protein. Green asterisks denote residues unique to *B*. *dolosa*.
(TIF)

S2 Fig *Burkholderia* species whole genome alignment surrounding and including the *lafA* gene region. The open reading frames of *B*. *dolosa* AU0158 are shown at the top. Regions of conservation (>70% sequence identity) in coding or intergenic regions of multiple *Burkholderia* species are indicated by purple and pink regions respectively. The alignment is based on pre-calculated values in the Integrated Microbial Genomes mVISTA portal.
(TIF)

S3 Fig Internalization of B. *dolosa* strains into host cells. *B*. *dolosa* wild-type and flagellin mutant strains were added to cultured RAW264.7 murine macrophage cultures at a M.O.I. of ~100 bacteria per host cell. Some cells were left un-infected as a negative control (“None”). The percentages of bacteria that invaded or were internalized by host cells were calculated from three independent replicates. Error bars represent one standard deviation of the data. One way ANOVA analysis gave an overall *p*-value of <0.0001 for the data. Asterisks above the bars indicate *p*-values of * < 0.05 or *** < 0.001 based on the Tukey’s multiple comparison test for all pairwise combinations.
(TIF)

S4 Fig Biofilm formation is not dependent on polar or lateral flagella. Strains were grown in 96-well PVC plates and assayed for biofilm growth using crystal violet staining after 4 days. Error bars represent one standard deviation of the data. An overall *p*-value of <0.0001 indicates that the means for each group are significantly different based on one-way ANOVA. Comparisons between groups were assessed using the Tukey multiple comparison test. ***—*p*-values <0.001.
(TIF)

S5 Fig Representative TEM images of *B*. *dolosa* wild-type and flagellin mutant strains. Four additional representative images for *B*. *dolosa* wild-type, Δ*fliC*, and Δ*lafA* in both stationary phase growth in liquid cultures or after growth on agar surfaces are shown. Scale bars are indicated on each panel.
(TIF)

S1 Table Genetic loci for the polar and putative lateral flagella of *B*. *dolosa* AU0158. Gene annotations for putative polar and lateral flagella are given in both the BDAG and AK34 designations. Genes are grouped by function based on other polar flagella systems. Genes not homologous to known flagellar proteins that were not found in the current B. dolosa annotations are listed as not found. Genes that appear to be present but are not currently annotated are listed as unannotated.
(PDF)

S2 Table Genes required for murine lung colonization based on Tn-seq analysis. A *B*. *dolosa* transposon mutant library was inoculated into the lungs of C57Bl/6 mice. After 48 hours, the library was harvested and junction fragments from input and output libraries were sequenced, mapped to the *B*. *dolosa* AU10158 genome, and normalized levels compared. Those with ≥ 2-fold changes and *q* values ≤ 0.05 are shown.
(PDF)

S3 Table Genes important for septic dissemination in a murine peritoneal model of infection. A *B*. *dolosa* transposon mutant library was inoculated into the peritoneum of C57Bl/6 mice. The library was harvested from the peritoneal cavity and the heart and junction fragments from input and output libraries were sequenced, mapped to the *B*. *dolosa* AU10158 genome, and normalized levels compared. Those with ≥ 2-fold changes and *q* values ≤ 0.05 are shown.
(PDF)
