# Supplementary figures and images for: A putative lateral flagella of the cystic fibrosis pathogen Burkholderia dolosa regulates swimming motility and host cytokine production
Source: PLoS One. 2018 Jan 18;13(1):e0189810. doi: 10.1371/journal.pone.0189810 (PMC5773237; doi:10.1371/journal.pone.0189810)

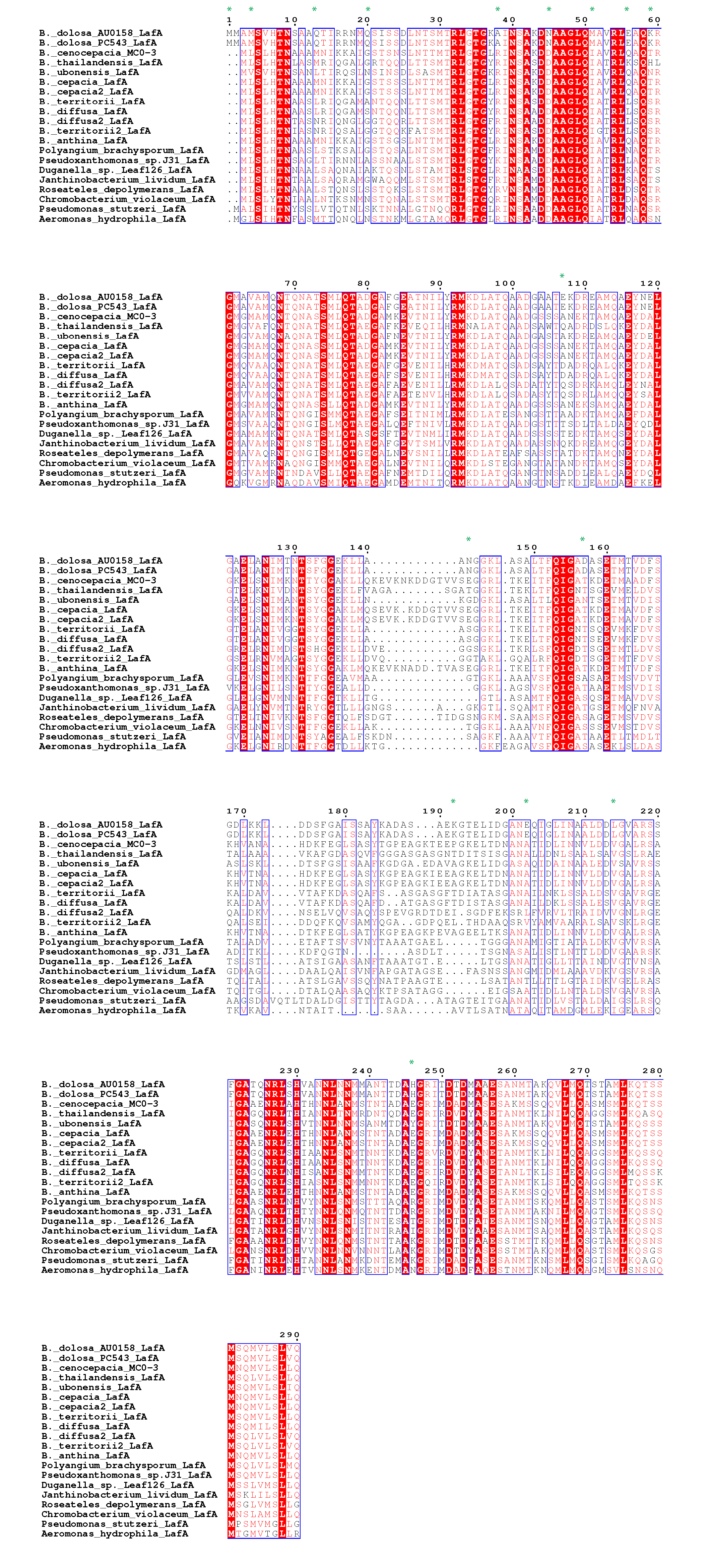

Supplement: S1 Fig — Amino acid sequences for lateral flagellins were obtained from GenBank and aligned using ClustalOmega. The resultant alignment was put into ESPript software to aid in visualization. The top three sequences belong to γ-proteobacteria species, the next 11 belong to Burkholderia strains and species, and the remaining six sequences belong to other β-proteobacteria species. Note that three Bcc species, B. cepacia, B. territorii, and B. diffusa, have two homologs of the LafA protein. Green asterisks denote residues unique to B. dolosa. (TIF) [file pone.0189810.s001.tif]

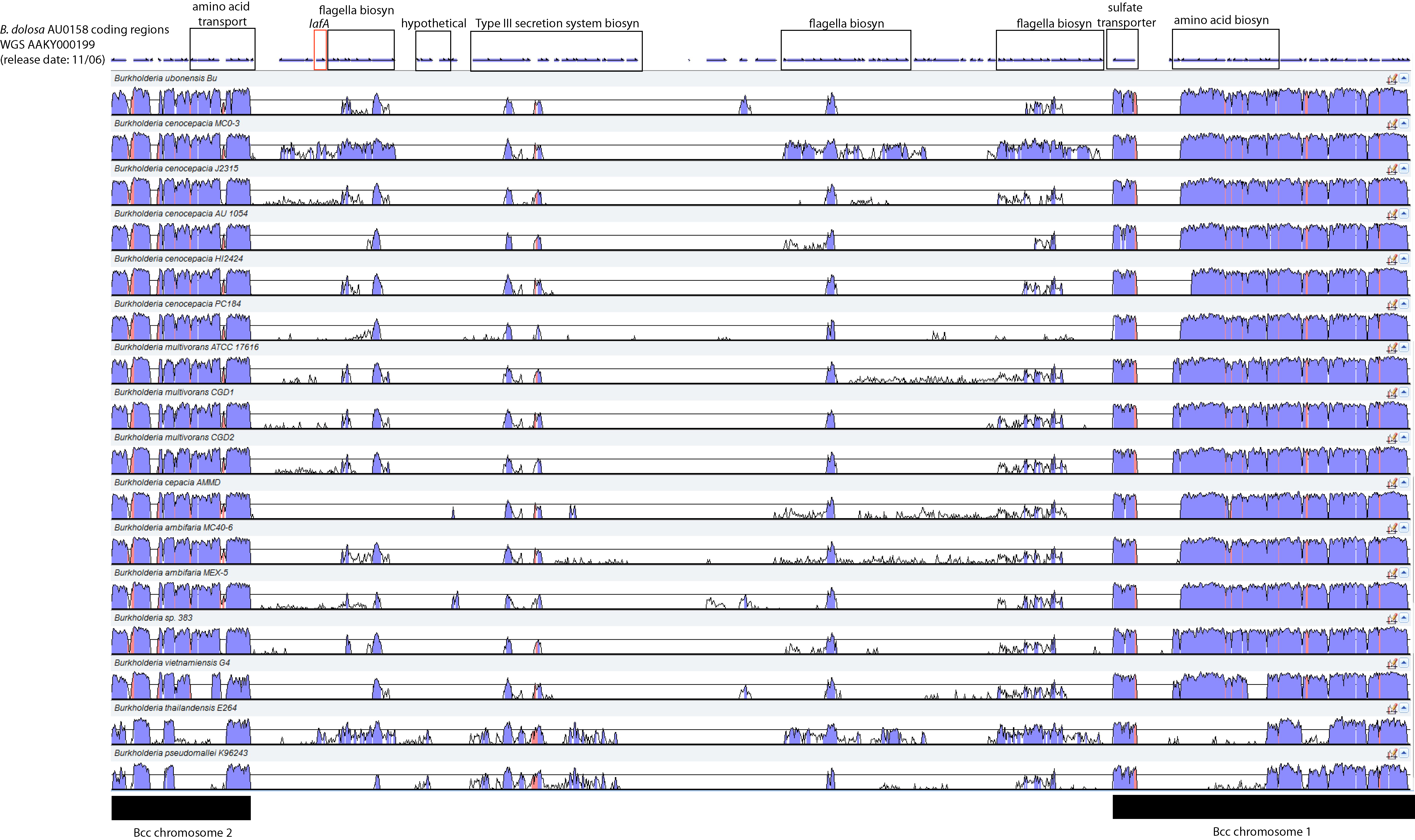

Supplement: S2 Fig — The open reading frames of B. dolosa AU0158 are shown at the top. Regions of conservation (>70% sequence identity) in coding or intergenic regions of multiple Burkholderia species are indicated by purple and pink regions respectively. The alignment is based on pre-calculated values in the Integrated Microbial Genomes mVISTA portal. (TIF) [file pone.0189810.s002.tif]

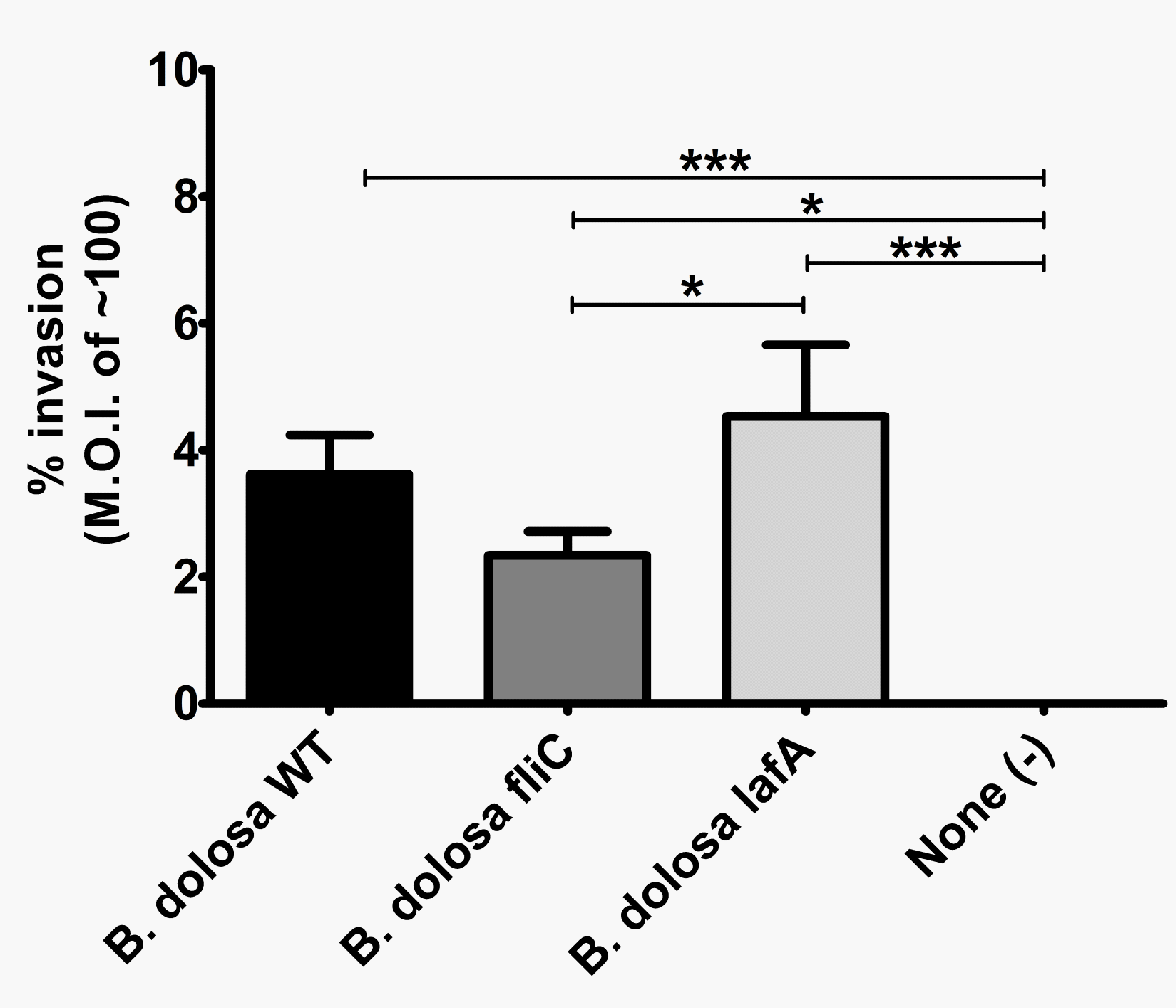

Supplement: S3 Fig — B. dolosa wild-type and flagellin mutant strains were added to cultured RAW264.7 murine macrophage cultures at a M.O.I. of ~100 bacteria per host cell. Some cells were left un-infected as a negative control (“None”). The percentages of bacteria that invaded or were internalized by host cells were calculated from three independent replicates. Error bars represent one standard deviation of the data. One way ANOVA analysis gave an overall p-value of <0.0001 for the data. Asterisks above the bars indicate p-values of * < 0.05 or *** < 0.001 based on the Tukey’s multiple comparison test for all pairwise combinations. (TIF) [file pone.0189810.s003.tif]

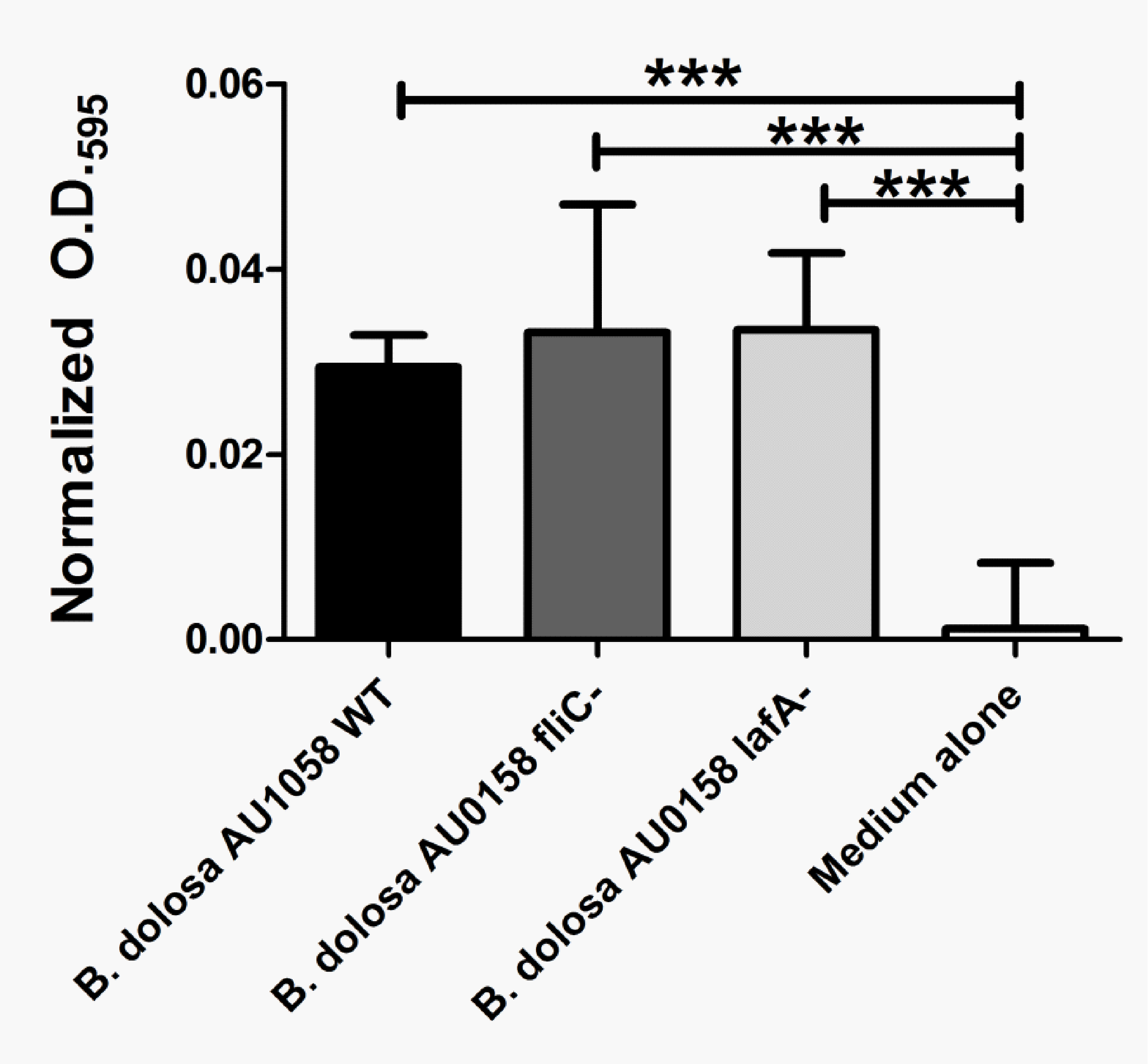

Supplement: S4 Fig — Strains were grown in 96-well PVC plates and assayed for biofilm growth using crystal violet staining after 4 days. Error bars represent one standard deviation of the data. An overall p-value of <0.0001 indicates that the means for each group are significantly different based on one-way ANOVA. Comparisons between groups were assessed using the Tukey multiple comparison test. ***—p-values <0.001. (TIF) [file pone.0189810.s004.tif]

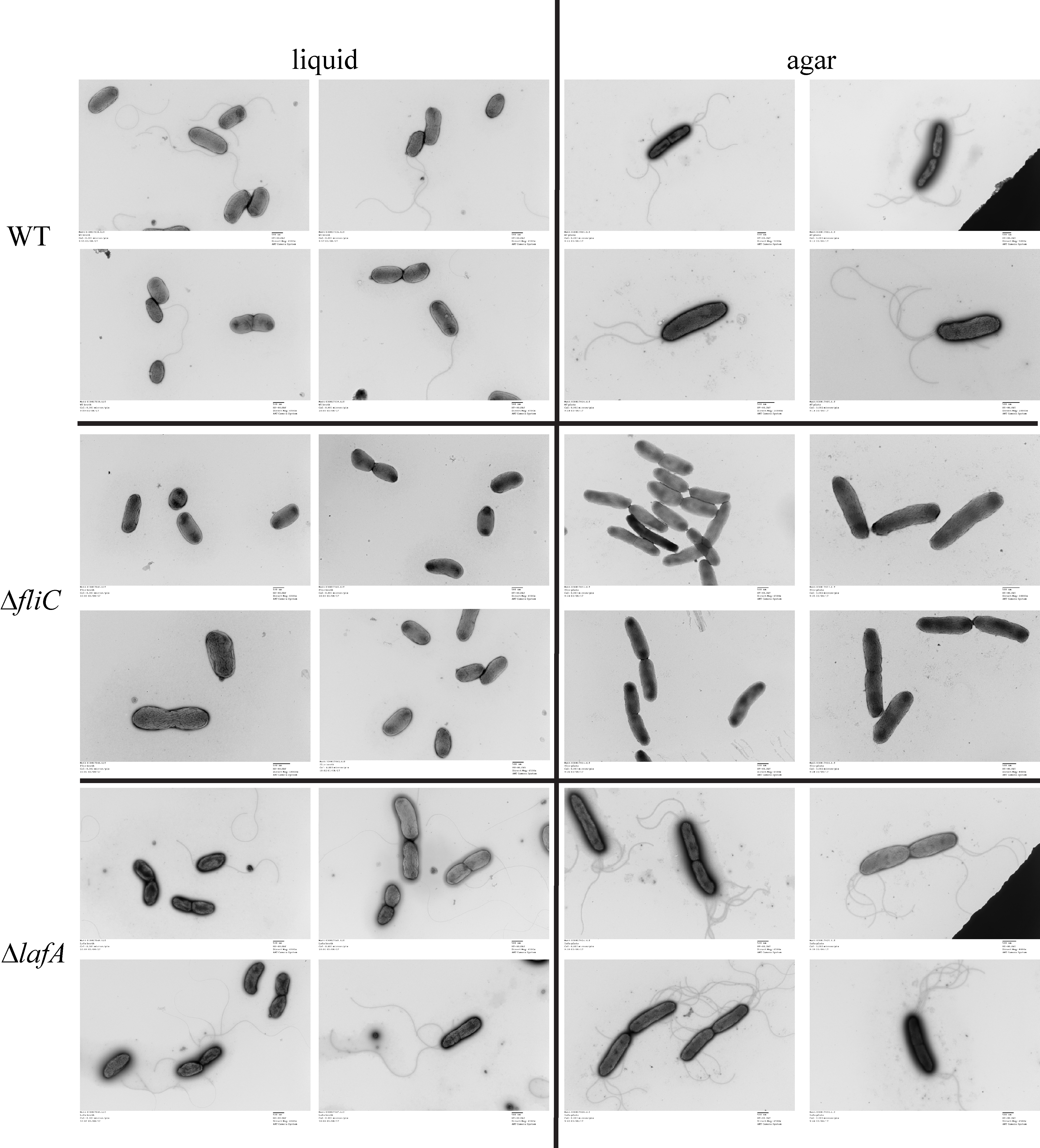

Supplement: S5 Fig — Four additional representative images for B. dolosa wild-type, ΔfliC, and ΔlafA in both stationary phase growth in liquid cultures or after growth on agar surfaces are shown. Scale bars are indicated on each panel. (TIF) [file pone.0189810.s005.tif]
