# Supplementary material for: A putative lateral flagella of the cystic fibrosis pathogen Burkholderia dolosa regulates swimming motility and host cytokine production
Source: PLoS One. 2018 Jan 18;13(1):e0189810. doi: 10.1371/journal.pone.0189810 (PMC5773237; doi:10.1371/journal.pone.0189810)
Supplement: S1 Table — Gene annotations for putative polar and lateral flagella are given in both the BDAG and AK34 designations. Genes are grouped by function based on other polar flagella systems. Genes not homologous to known flagellar proteins that were not found in the current B. dolosa annotations are listed as not found. Genes that appear to be present but are not currently annotated are listed as unannotated. (PDF) [file pone.0189810.s006.pdf]

Table S1. Genetic loci for the polar and putative lateral flagella of *B. dolosa* AU0158

		Polar flagella*		Lateral flagella*	
**Gene Name**	**Annotation**	**Broad Designation**	**Published sequence**	**Broad Designation**	**Published sequence**
Flagellar export, assembly, and switching					
fliL	flagellar basal body-associated protein	BDAG_00198	AK34_RS28135	not found	not found
fliM	flagellar motor switch protein	BDAG_00199	AK34_RS28140	BDAG_04367	AK34_RS07890
fliN	Flagellar motor switch	BDAG_00200	AK34_RS28145	BDAG_00331	AK34_RS07885
fliO	flagellar export pore protein	BDAG_00201	AK34_RS28150	not found	not found
fliP	flagellar export pore protein	BDAG_00202	AK34_RS28155	BDAG_04368	AK34_RS07880
fliQ	flagellar export pore protein	BDAG_00203	AK34_RS28160	not annotated	AK34_RS07875
fliR	flagellar export pore protein	BDAG_00204	AK34_RS28165	BDAG_04369	AK34_RS07870
flhF	flagellar localization regulator	BDAG_00056	AK34_RS27355	not found	not found
flhG	flagellar localization regulator	not annotated	AK34_RS27350	not found	not found
Flagellar hook apparatus					
flgN	FlgK and FlgL chaperone	BDAG_00361	AK34_RS13405	BDAG_04404	AK34_RS28780
flgA	flagellar basal body P-ring biosynthesis protein	BDAG_00363	AK34_RS13415	not annotated	AK34_RS07570
flgB	flagellar basal body rod protein	BDAG_00364	AK34_RS13420	BDAG_04410	AK34_RS07565
flgC	flagellar basal body rod protein	BDAG_00365	AK34_RS13425	BDAG_04411	AK34_RS07560
flgD	flagellar hook cap	BDAG_00366	AK34_RS13430	BDAG_04412	AK34_RS07555
flgE	flagellar hook protein	BDAG_00367	AK34_RS13435	BDAG_04413	AK34_RS07550
flgF	flagellar basal body rod protein	BDAG_00368	AK34_RS13440	BDAG_04414	AK34_RS07545
flgG	flagellar basal body distal rod	BDAG_00369	AK34_RS13445	BDAG_04415	AK34_RS07540
flgH	flagellar basal body L-ring protein	BDAG_00370	AK34_RS13450	BDAG_04416	AK34_RS07535
flgI	flagellar basal body P-ring protein	BDAG_00371	AK34_RS13455	BDAG_04417	AK34_RS07530
flgJ	peptidoglycan hydrolase	BDAG_00372	AK34_RS13460	unannotated^	AK34_RS07525
flgK	flagellar hook-associated protein	BDAG_00374	AK34_RS13470	BDAG_04418	unannotated‡
flgL	flagellar hook-associated protein	BDAG_00375	AK34_RS13475	BDAG_04419	AK34_RS07515
Flagellar basal body					
fliE	flagellar hook-basal body complex protein	not annotated	AK34_RS13235	BDAG_04392	AK34_RS07680
fliF	flagellar MS-ring protein	BDAG_00332	AK34_RS13230	BDAG_04393	AK34_RS07675
fliG	flagellar motor switch protein	BDAG_00331	AK34_RS13225	BDAG_00331	AK34_RS07670
fliH	flagellar protein export	BDAG_00330	AK34_RS13220	BDAG_04395	AK34_RS07665
fliI	flagellar protein export ATPase	BDAG_00329	AK34_RS13215	BDAG_04396	AK34_RS07660
fliJ	flagellar protein export	BDAG_00328	AK34_RS13210	BDAG_04397	AK34_RS07655
ycgR	flagellar brake protein	BDAG_00373	AK34_RS13465	not found	not found
fliK	flagellar hook-length control protein	BDAG_00327	AK34_RS13205	not annotated	AK34_RS07635
flhA	flagellar export pore protein	BDAG_00057	AK34_RS27360	BDAG_04371	AK34_RS07860
flhB	flagellar export pore protein	BDAG_00058	AK34_RS27365	BDAG_04370	AK34_RS07865
fliS	FliC-specific chaperone	not annotated	AK34_RS13240	BDAG_04398	AK34_RS07645
fliT	FliD-specific chaperone	not annotated	AK34_RS13245	not found	not found
Flagellar expression					

fliA	flagellar biosynthesis sigma factor	BDAG_00055	AK34_RS27345	BDAG_04400	AK34_RS07625
flgM	anti-sigma factor for flagella	BDAG_00362	AK34_RS13410	BDAG_04403	AK34_RS07610
flhC	transcription of flagellar genes	BDAG_00073	AK34_RS27445	not found	not found
flhD	transcription of flagellar genes	BDAG_00074	AK34_RS27450	not found	not found
Flagellar rotation					
motA	flagellar motor protein	BDAG_00072	AK34_RS27440	BDAG_04401	AK34_RS07620
motB	flagellar motor protein	BDAG_00071	AK34_RS27435	BDAG_04402	AK34_RS03990, AK34_RS11495
fliL	flagellar rotation protein	not found	not found	BDAG_04399	AK34_RS07630
Flagellar structure and length					
fliC	flagellin	BDAG_00084	AK34_RS27500	BDAG_04366	AK34_RS07895
fliD	flagellar hook-associated protein	BDAG_00085	AK34_RS27505	Unannotated[†]	AK34_RS07650
fliB	Flagellin lysine methylase	not found	not found	not found	not found

[*]ORF designations given by the Broad Institutes annotation (http://www.broadinstitute.org/scientific-community/data/burkholderia-dolosa) or that published in [1]. Not found indicates that no genetic loci can be identified that matches known sequences for this gene or the predicted protein sequence. Not annotated indicates that this gene was annotated in only one version of the genome.

[^] Unannotated ORF located between BDAG_04417 and BDAG_04418 found

[‡] Unannotated ORF located between AK34_RS07515 and AK34_RS 07525

[†] Unannotated ORF located between BDAG_04398 and BDAG_004399
