# Supplementary material for: A putative lateral flagella of the cystic fibrosis pathogen Burkholderia dolosa regulates swimming motility and host cytokine production
Source: PLoS One. 2018 Jan 18;13(1):e0189810. doi: 10.1371/journal.pone.0189810 (PMC5773237; doi:10.1371/journal.pone.0189810)
Supplement: S2 Table — A B. dolosa transposon mutant library was inoculated into the lungs of C57Bl/6 mice. After 48 hours, the library was harvested and junction fragments from input and output libraries were sequenced, mapped to the B. dolosa AU10158 genome, and normalized levels compared. Those with ≥ 2-fold changes and q values ≤ 0.05 are shown. (PDF) [file pone.0189810.s007.pdf]

Table S2. Genes required for murine lung colonization based on Tn-seq analysis

Query locus tag (BDAG_0)	Annotation	Lung colonization model
0002	phenylacetate-CoA oxygenase subunit PaaA	-2.85
0003	phenylacetate-CoA oxygenase subunit PaaB	-2.56
0004	phenylacetate-CoA oxygenase PaaI	-2.46
0005	phenylacetate-CoA oxygenase PaaJ	-2.25
0006	Phenylacetate-CoA oxygenase/reductase, PaaK	-2.89
0052	5,10-methylenetetrahydrofolate reductase	-6.53
0053	hypothetical protein	-3.72
0057	flagellar biosynthesis protein FlhA	2.39
0058	flagellar biosynthesis protein FlhB	2.40
0073	transcriptional activator FlhC	2.97
0074	transcriptional activator FlhD	2.83
0102	UvrD/REP helicase	-2.61
0174	sodium ion exchange antiporter	2.02
0201	flagellar protein FliO	2.44
0202	flagellar biosynthesis protein FliP	2.32
0203	flagellar biosynthesis protein FliQ	2.97
0204	flagellar biosynthetic protein FliR	2.54
0229	hypothetical protein	4.27
0244	AAA family ATPase protein	-2.45
0252	peptide deformylase	-2.79
0268	endonuclease/exonuclease/phosphatase family protein	-2.00
0274	homoserine O-acetyltransferase	-8.03
0301	acetylglutamate kinase	-2.89
0327	flagellar hook-length control protein FliK	2.57
0328	flagellar FliJ protein	2.79
0329	flagellum-specific ATP synthase FliI	2.34
0330	flagellar assembly protein H	2.54
0331	flagellar motor switch protein G	2.07
0332	flagellar MS-ring protein	2.69
0336	two-component regulatory system, response regulator protein	3.24
0337	sensor kinase protein	3.77
0363	flagellar basal body P-ring biosynthesis protein FlgA	2.62
0364	flagellar basal body rod protein FlgB	2.58
0365	flagellar basal body rod protein FlgC	2.73
0366	flagellar basal body rod modification protein	2.45
0367	flagellar hook protein FlgE	2.35
0368	flagellar basal body rod protein FlgF	2.06
0369	flagellar basal body rod protein FlgG	2.60
0370	flagellar basal body L-ring protein	2.41
0371	flagellar basal body P-ring protein	2.44
0372	flagellar rod assembly protein/muramidase FlgJ	2.12
0392	putrescine ABC transporter permease	2.57

0445	biotin synthase	-2.63
0447	8-amino-7-oxononanoate synthase	-2.16
0448	adenosylmethionine--8-amino-7-oxononanoate transaminase	-2.34
0459	sporulation domain-containing protein	-2.77
0460	arginyl-tRNA synthetase	3.11
0501	thiol-disulfide oxidoreductase	-3.62
0513	phosphoglyceromutase	-2.33
0516	preprotein translocase subunit SecB	2.76
0524	cytochrome c oxidase polypeptide I	-2.82
0571	P-loop ATPase family protein	-7.23
0575	RNA polymerase factor sigma-, rpoN1	-2.37
0588	major facilitator superfamily protein	-3.04
0606	MarR family regulatory protein	-2.53
0613	threonine dehydratase	-3.12
0614	oxidoreductase	-4.99
0627	acyl-CoA dehydrogenase oxidoreductase protein	-2.22
0628	3-hydroxyacyl-CoA dehydrogenase oxidoreductase	-2.21
0644	acyltransferase	-4.30
0676	periplasmic cytochrome c containing protein	-2.10
0700	O-antigen polymerase family protein	-7.70
0725	two-component regulatory system, response regulator protein	-8.21
0727	subfamily S1B serine peptidase	2.37
0767	cyclopropane-fatty-acyl-phospholipid synthase	-10.65
0773	N-acetylmuramoyl-L-alanine amidase	-9.61
0783	ornithine carbamoyltransferase	-8.05
0805	hypothetical protein	-2.00
0808	isocitrate dehydrogenase	-2.25
0810	ATP-dependent Clp protease adaptor protein ClpS	-3.31
0811	ATP-dependent Clp protease ATP-binding subunit	-5.16
0818	phosphoribosylglycinamide formyltransferase	-2.45
0861	cytochrome c protein	7.07
0867	cell division protein ZapA	-6.13
0889	superoxide dismutase SodC	-2.09
0891	ornithine decarboxylase	-14.51
0892	Lysine decarboxylase	-3.94
0900	phosphoenolpyruvate carboxylase	-2.29
0910	NAD synthetase	3.11
0917	histidine transport system permease	2.06
0961	DNA topoisomerase IV subunit B	-5.18
0962	DNA topoisomerase IV subunit A	-8.63
0982	transaldolase B	-2.30
0986	cytochrome C -related protein	-2.71
0990	endonuclease III	3.28
1005	Lipid A core - O-antigen ligase	-4.16
1006	glycosyl transferase family protein	-2.88
1011	major facilitator superfamily mfs_1	3.07

1016	iojap family protein	-2.45
1028	hypothetical protein	13.46
1050	acetolactate synthase 3 regulatory subunit	-8.46
1077	hypothetical protein	-3.74
1102	excinuclease ABC subunit B	-4.14
1143	N-acetylglutamate synthase	-6.37
1155	endonuclease/exonuclease/phosphatase family protein	-2.28
1178	2Fe-2S ferredoxin	-7.41
1192	phosphopyruvate hydratase	-3.45
1219	HhH-GPD superfamily base excision DNA repair protein	-2.17
1264	ZipA FtsZ-binding region	-3.81
1268	PII uridylyl-transferase	-2.17
1277	protease EcfE	-8.85
1285	RNA methylase protein	2.20
1287	phosphoenolpyruvate synthase	-3.25
1296	hypothetical protein	2.99
1348	penicillin-binding protein	-2.67
1370	trigger factor	7.46
1371	ATP-dependent Clp protease proteolytic subunit	-27.50
1372	ATP-dependent protease ATP-binding subunit ClpX	-3.48
1373	ATP-dependent protease La	-19.66
1393	stress responsive A/B barrel domain protein	-2.12
1395	lipase	2.07
1418	putative transmembrane anti-sigma factor	-2.43
1467	L-isoaspartate O-methyltransferase	-4.13
1468	family M23 peptidase	-24.92
1469	RNA polymerase sigma factor RpoS	-2.29
1486	hypothetical protein	5.54
1487	ATP phosphoribosyltransferase regulatory subunit	-8.20
1492	Lipoprotein	-3.03
1494	ATP-dependent DNA helicase-related protein	-2.05
1501	polyhydroxyalkanoate (PHA) synthesis regulatory protein	-2.58
1507	phosphoserine phosphatase	-10.10
1519	outer membrane protein	-2.90
1529	hypothetical protein	2.41
1534	TetR family regulatory protein	-2.62
1542	LacI family regulatory protein	-3.64
1543	hypothetical protein	5.00
1564	MarR family regulatory protein	-2.11
1603	hypothetical protein	-4.62
1625	ribose operon repressor	-2.11
1634	LexA repressor	-55.85
1745	membrane protein	4.31
1747	lipoprotein	4.79
1748	flp type pilus assembly protein	3.39
1749	flp type pilus assembly protein	6.06

1750	flp pilus type assembly protein	8.42
1751	flp pilus type assembly-related protein	2.17
1772	Ribosomal large subunit pseudouridine synthase B	-4.81
1785	MerR family regulatory protein	-2.22
1797	hypothetical protein	3.11
1810	XRE family transcriptional regulator	-3.17
1895	signal transduction histidine kinase with and activity	2.47
1933	phosphate transport system-related protein	-3.18
1934	phosphate transporter ATP-binding protein	-3.14
1935	phosphate transporter permease subunit PtsA	-2.39
1936	phosphate transporter permease subunit PstC	-2.67
1937	phosphate transport system substrate-binding exported periplasmic protein	-2.63
1946	leucine export protein LeuE	-2.86
1947	carbamoyl phosphate synthase small subunit CarA	-21.12
1950	gultathione hydrolase	-2.10
1951	methyltransferase	-7.96
2025	arginine N-succinyltransferase subunit alpha	-10.33
2026	bifunctional N-succinyldiaminopimelate-aminotransferase/acetylornithine transaminase protein	-2.77
2037	hypothetical protein	-4.29
2038	YdjC-like protein	-4.95
2041	luciferase-like monooxygenase	3.40
2047	GntR family regulatory protein	-2.01
2087	DNA repair protein RecO	-2.50
2094	sigma-E factor regulatory protein RseB	-2.08
2096	RNA polymerase sigma factor RpoE	-36.60
2114	molybdenum cofactor biosynthesis protein A	-2.31
2173	30S ribosomal protein S1	2.29
2192	hypothetical protein	-4.31
2241	DNA translocase FtsK	-3.05
2251	bifunctional glucokinase/RpiR family transcriptional regulator	-3.43
2254	glutamate-1-semialdehyde aminotransferase	11.45
2263	3-octaprenyl-4-hydroxybenzoate carboxy-lyase	-4.41
2266	hypothetical protein	-2.78
2293	urease subunit gamma	3.12
2303	O-antigen exporter	-38.34
2304	glycosyltransferase	-10.67
2305	glycosyltransferase	-14.24
2306	UDP-glucose epimerase	-12.30
2307	UDP-N-acetylglucosamine-1-P transferase	-17.67
2311	UbiA prenyltransferase	-2.96
2314	NAD-dependent epimerase/dehydratase	-2.67
2315	Amine oxidase:FAD dependent oxidoreductase	-3.29
2318	group 1 family protein	-2.90
2319	nad-dependent epimerase dehydratase family protein	-2.02
2321	group 1 glycosyl transferase	-2.43

2322	methyltransferase type 11	-2.43
2324	dTDP-4-keto-L-rhamnose reductase	-4.15
2325	dTDP-4-keto-6-deoxy-D-glucose 3,5-epimerase	-9.90
2326	glucose-1-phosphate thymidylyltransferase	-4.81
2330	ABC transporter ATP-binding protein	-6.88
2331	ABC-2 type transporter	-2.16
2340	rubredoxin	-4.93
2346	hypothetical protein	3.39
2347	putative transmembrane anti-sigma factor	-2.97
2395	TolQ transport transmembrane protein	-2.26
2397	possible TolA-related transport transmembrane protein	-6.28
2398	translocation protein TolB	-13.34
2399	OmpA family lipoprotein	-31.50
2400	tol-pal system protein YbgF	-2.37
2405	DedA family protein	-2.17
2408	tRNA delta(2)-isopentenylpyrophosphate transferase	-9.70
2412	HAD family hydrolase	-13.99
2413	polynucleotide adenylyltransferase	-2.21
2417	chorismate binding enzyme	-2.22
2425	heat-inducible transcription repressor	-2.31
2433	phospho-2-dehydro-3-deoxyheptonate aldolase	-2.51
2442	pyrroline-5-carboxylate reductase	-4.05
2446	oxidative stress regulatory protein	-7.76
2475	DNA-binding protein Fis	-2.46
2495	LysR family regulatory protein	-3.44
2512	dihydrodipicolinate reductase	-2.56
2526	barstar family protein	-2.57
2527	exported ribonuclease	-2.25
2529	Hypothetical protein	9.51
2547	shikimate 5-dehydrogenase	-3.25
2559	sugar kinase	-2.72
2563	N-acetyl-anhydromuranmyl-L-alanine amidase	-2.05
2564	cytochrome c assembly protein	-4.18
2571	gamma-glutamyl kinase	-2.79
2583	bifunctional ornithine acetyltransferase/N-acetylglutamate synthase protein	-2.07
2598	peptidoglycan synthetase FtsI	-2.80
2612	outer membrane-bound lytic murein transglycosylase	-2.89
2614	ribulose-phosphate 3-epimerase	-4.83
2616	anthranilate synthase component I	-7.99
2617	anthranilate synthase component II	-10.85
2618	anthranilate phosphoribosyltransferase	-9.39
2619	indole-3-glycerol-phosphate synthase	-4.64
2640	glutaredoxin	-3.67
2641	3-octaprenyl-4-hydroxybenzoate carboxy-lyase	-4.21
2650	hypothetical protein	3.17
2684	phage resolvase/recombinase for integration and excision	4.17

2706	phosphoribosyl-ATP pyrophosphatase	-4.43
2707	phosphoribosyl-AMP cyclohydrolase	-5.41
2708	imidazole glycerol phosphate synthase subunit HisF	-10.33
2709	1-(5-phosphoribosyl)-5-[(5- phosphoribosylamino)methylideneamino] imidazole-4-carboxamide isomerase	-10.08
2710	imidazole glycerol phosphate synthase subunit HisH	-11.35
2712	imidazoleglycerol-phosphate dehydratase	-16.00
2714	histidinol dehydrogenase	-12.00
2715	ATP phosphoribosyltransferase catalytic subunit	-10.39
2716	UDP-N-acetylglucosamine 1-carboxyvinyltransferase	-3.33
2717	BolA-like protein	-4.13
2718	ABC transporter membrane protein	-3.28
2719	ABC transporter ATP-binding protein	-3.33
2732	glutamate synthase subunit beta	-4.85
2733	glutamate synthase large subunit	-4.41
2734	hypothetical protein	-2.75
2746	penicillin-binding protein 1A	-2.75
2829	glycosyltransferase	6.82
2848	ABC transporter ATP-binding protein	-2.44
2917	hypothetical protein	3.24
2924	isopropylmalate isomerase large subunit	-5.71
2925	isopropylmalate isomerase small subunit	-6.06
2926	3-isopropylmalate dehydrogenase	-5.92
2928	Tfp pilus assembly protein FimV	-2.38
2930	N-(5'-phosphoribosyl)anthranilate isomerase	-10.22
2931	tryptophan synthase subunit beta	-8.52
2932	tryptophan synthase subunit alpha	-3.46
2934	bifunctional folylpolyglutamate synthase/dihydrofolate synthase	-7.62
2935	peptidoglycan-binding membrane protein	-2.55
2936	colicin V production protein	-2.52
2937	amidophosphoribosyltransferase	-9.93
2951	porin	4.91
3035	TetR family transcriptional regulator	-3.77
3036	hypothetical protein	2.79
3097	cysteine peptidase/transferase, family C45	2.82
3124	hypothetical protein	4.98
3205	penicillin-binding protein	4.38
3207	NAD dependent epimerase/dehydratase	-2.24
3253	IclR family regulatory protein	2.47
3315	hypothetical protein	2.95
3331	periplasmic sensor signal transduction histidine kinase	2.14
3365	type vi secretion system hcp1 family	2.67
3405	hypothetical protein	2.25
3538	porin	5.75
3609	hypothetical protein	-2.56
3664	diaminopimelate decarboxylase	-4.41

3702	two-component regulatory system sensor kinase protein	-3.20
3779	amine dehydrogenase	2.96
3787	choline-sulfatase	2.69
3874	diguanylate phosphodiesterase	2.00
4007	beta-lactamase family protein	3.06
4030	cation-transporting ATPase membrane protein	-8.12
4061	oligopeptide ABC transporter ATP-binding protein	2.30
4138	Toluene tolerance	-3.26
4142	protein	3.04
4349	argininosuccinate synthase	-3.14
4359	glycosyltransferase	3.61
4411	flagellar basal-body rod protein	2.42
4665	major facilitator superfamily protein	-5.36
4666	LysR family transcriptional regulator	-6.90
4687	hypothetical protein	6.85
4744	GNAT family acetyltransferase	2.66
4771	benzoate 1,2-dioxygenase alpha subunit	2.23
4979	exported glyoxalase	2.07
4986	DeoR family regulatory protein	-2.37
4996	two-component regulatory system, response regulator protein	2.44
5003	peptide methionine sulfoxide reductase family protein	3.04
5008	short-chain dehydrogenase family protein	-2.50
newgene_132	pilus assembly protein (cpaC)	5.19
