# Supplementary material for: A putative lateral flagella of the cystic fibrosis pathogen Burkholderia dolosa regulates swimming motility and host cytokine production
Source: PLoS One. 2018 Jan 18;13(1):e0189810. doi: 10.1371/journal.pone.0189810 (PMC5773237; doi:10.1371/journal.pone.0189810)
Supplement: S3 Table — A B. dolosa transposon mutant library was inoculated into the peritoneum of C57Bl/6 mice. The library was harvested from the peritoneal cavity and the heart and junction fragments from input and output libraries were sequenced, mapped to the B. dolosa AU10158 genome, and normalized levels compared. Those with ≥ 2-fold changes and q values ≤ 0.05 are shown. (PDF) [file pone.0189810.s008.pdf]

Table S3. Genes important for septic dissemination in a murine peritoneal model of infection

Locus (BDAG_0)	Annotation	Fold Change Peritoneum	Fold Change Heart
0035	hypothetical protein	2.68	
0036	cytochrome c551/c552	-4.56	-6.06
0041	metallo-beta-lactamase superfamily protein	3.28	
0045	histone-like nucleoid-structuring (H-NS) protein		-5.50
0065	response regulator receiver modulated CheB methylesterase	4.75	
0073	transcriptional activator FlhC	3.94	
0074	transcriptional activator FlhD	4.85	
0111	conserved hypothetical protein	-2.13	
0163	AnsC family regulatory protein		-55.39
0170	NADH-dependent flavin oxidoreductase	2.23	
0212	transmembrane regulator		-18.00
0258	hypothetical protein	-3.20	-8.24
0262	rod shape-determining protein MreC	-2.94	
0263	rod shape-determining protein MreB	-5.22	
0268	endonuclease/exonuclease/phosphatase family protein	-3.26	-7.55
0329	flagellum-specific ATP synthase FliI	2.36	
0330	flagellar assembly protein H		2.47
0336	LuxR-family response regulator		2.95
0337	histidine kinase protein	2.93	
0355	MarR family regulatory protein		-4.59
0373	YcgR family protein		-3.41
0523	cytochrome c oxidase	-2.93	
0524	cytochrome c oxidase polypeptide I	-2.05	-3.26
0529	hypothetical protein	-2.62	
0540	ABC ATP-binding protein	5.41	
0552	RNA polymerase factor sigma-32	-9.21	-12.65
0560	phosphopantetheine adenylyltransferase	-12.81	-7.18
0625	phospholipase D	-2.26	
0694	recombination regulator RecX	-2.46	-2.41
0727	subfamily S1B serine peptidase	-9.83	-5.41
0730	carbon monoxide dehydrogenase	3.71	
0755	DeoR family transcriptional regulator		-2.66
0778	AMP-binding enzyme	2.11	
0808	isocitrate dehydrogenase		-4.52

0824	cell envelope biogenesis protein OmpA	-4.55	
0872	cobalamin synthase	3.04	
0873	alpha-ribazole phosphatase	2.72	
0891	ornithine decarboxylase	-5.55	-7.64
0895	transposase	-5.30	-8.51
0911	nitrogen regulatory protein P-II 2	-5.99	-12.74
0934	short chain dehydrogenase	5.40	
1021	uracil phosphoribosyltransferase	-2.51	-3.01
1050	acetolactate synthase 3 regulatory subunit	-2.53	
1085	glutathione S-transferase	-2.54	
1123	haloacid dehalogenase-like hydrolase		-3.62
1166	D-alanyl-D-alanine endopeptidase		-4.88
1192	phosphopyruvate hydratase	-2.38	-4.16
1196	anhydrase	-5.84	
1200	translation factor Sua5	-2.09	
1202	tryptophanyl-tRNA synthetase		-4.16
1238	gamma-glutamyl cyclotransferase	2.44	
1243	hypothetical protein	-2.33	
1283	hypothetical protein	-5.37	-3.95
1334	polysaccharide deacetylase	-3.23	
1335	uricase		-7.27
1351	acetylornithine deacetylase	-2.60	
1372	ATP-dependent protease ATP-binding subunit ClpX	-4.19	
1400	thymidylate kinase	-4.00	
1469	RNA polymerase sigma factor RpoS	-4.64	-8.55
1501	polyhydroxyalkanoate (PHA) synthesis regulatory protein	-5.08	
1509	NADPH:quinone reductase	4.73	
1550	metal ion transport protein	-2.13	
1553	hypothetical protein	-3.43	
1556	(R)-stereoselective amidase	-2.82	-4.12
1622	PrkA family serine protein kinase		-2.12
1625	ribose operon repressor	-1.91	-2.35
1639	hypothetical protein	5.06	
1700	hypothetical bacteriophage protein	-2.42	
1757	transposase		-7.97
1763	MarR family regulatory protein	-4.27	
1773	SMC-Scp complex subunit ScpB	-24.11	-7.75
1788	phenylalanyl-tRNA synthetase subunit alpha		-9.87
1842	ribose ABC transporter substrate-binding exported	-5.47	-4.03

	protein		
1863	hypothetical protein	3.18	
1877	pirin-like protein	6.03	
1903	hypothetical protein	2.32	
1927	carbamoyl phosphate synthase small subunit	-2.27	
1937	phosphate transport system substrate-binding exported periplasmic protein	-2.86	
1949	exported transglycosylase protein	-2.34	-3.36
1972	LysR family regulatory protein		-4.80
2045	phospholipase phosphocholine-specific	10.04	
2050	membrane protein	-2.56	
2080	excinuclease ABC subunit c	-3.05	-5.03
2092	glutaredoxin	3.34	
2173	30S ribosomal protein S1	-4.09	-3.19
2194	general substrate transporter	2.13	
2214	cold shock-like protein	-5.46	
2235	septum site-determining protein		-3.21
2251	bifunctional glucokinase/RpiR family transcriptional regulator	-2.39	-4.92
2295	urease subunit alpha	-2.48	-3.45
2306	UDP-glucose epimerase		-15.56
2321	group 1 glycosyl transferase		-4.21
2340	rubredoxin		-91.44
2342	chaperonin GroEL	-3.80	-5.11
2362	polyketide cyclase		-3.87
2384	hypothetical protein	-4.26	
2400	Tol-Pal system protein YbgF	-2.57	-3.07
2405	DedA family protein	-4.20	-14.08
2416	3-methyl-2-oxobutanoate hydroxymethyltransferase	-5.82	-5.55
2450	preprotein translocase subunit YajC	-11.64	-11.03
2493	periplasmic glutamate/aspartate-binding protein		-3.29
2494	glutamate dehydrogenase	-4.56	-4.70
2511	transport-related membrane protein	2.15	
2530	phosphatidylglycerophosphatase		-4.36
2533	orotidine 5'-phosphate decarboxylase	-5.27	-25.69
2555	acetyl-CoA carboxylase biotin carboxylase subunit	-3.57	-2.38
2559	sugar kinase	-2.25	-6.45
2578	Type II secretion system protein	2.73	
2633	hydrolase	2.22	
2659	outer membrane protein	-4.48	

2664	type VI secretion system protein TssE		-3.45
2715	ATP phosphoribosyltransferase catalytic subunit	-8.72	-22.25
2742	3-dehydroquinate synthase	-4.13	
2753	cytochrome c4	-12.52	-12.16
2755	delta-aminolevulinic acid dehydratase	-3.47	-2.49
2756	thiol:disulfide interchange protein DsbD	-3.09	
2757	divalent-cation tolerance protein CutA	-2.02	-3.41
2758	50S ribosomal protein L17	-200.50	-2.90
2806	phosphoesterase	4.05	
2869	geranyltranstransferase		-6.04
2882	major facilitator superfamily protein		-2.20
2883	efflux system transport protein	2.74	
2935	peptidoglycan-binding membrane protein		-4.53
2946	hypothetical protein		-6.99
2957	FdhE-like protein	2.90	
2960	methionyl-trna formyltransferase	2.89	
2974	type ii secretion system protein e		-8.12
3010	LysR family regulatory protein		-4.60
3035	TetR family transcriptional regulator	-11.17	-8.88
3057	hypothetical protein		-9.41
3107	nitrogen assimilation regulatory protein Nac	2.35	
3157	peptidase		3.60
3183	drug:proton antiporter	2.69	
3192	Crp/Fnr family transcriptional regulator		-6.82
3270	LysR family regulatory protein	-2.62	
3330	cation efflux protein	-6.49	
3425	fumarylpyruvate hydrolase	7.84	
3440	MarC family protein	-7.47	
3482	ABC transporter ATP-binding protein	-4.05	
3583	thioesterase superfamily protein	-2.56	-26.21
3587	sulfonate ABC transporter permease	-10.59	-8.06
3609	oxidoreductase	-5.01	
3648	type III secretion system protein SctS	2.05	
3650	LuxR superfamily regulatory protein		-3.82
3661	membrane protein	-9.59	
3681	NnrU family protein		-3.48
3686	transcriptional activator FtrA	-1.78	-2.91
3701	patatin-like phospholipase	-1.72	-2.17
3800	hypothetical protein	-3.24	

3845	HPr kinase	2.42	
3874	diguanylate phosphodiesterase		4.61
3891	inosine-uridine preferring nucleoside hydrolase	3.42	
3968	IclR family regulatory protein	-2.76	
3986	TetR family regulatory protein		-6.73
4008	glutathione S-transferase	-2.12	
4014	ArsR family regulatory protein	-2.15	
4022	hypothetical protein	-2.12	
4159	hypothetical protein	-14.53	
4167	50S ribosomal protein L21	2.05	
4178	transporter-LysE family		-3.31
4204	LysR family regulatory protein	-5.67	-5.51
4220	lysine-arginine-ornithine transport binding exported protein		-3.89
4266	polypeptide-transport-associated domain-containing protein	-2.12	
4336	hypothetical protein		-3.48
4424	LysE type translocator	-2.33	
4462	phage integrase family protein	2.24	
4473	hypothetical protein	-5.48	
4476	hydrolase		-4.82
4532	LysR family regulatory protein		-5.52
4543	C4-dicarboxylate ABC transporter permease	4.30	
4589	Flp type pilus assembly protein		-6.22
4606	ABC transporter ATP-binding protein	-2.22	
4608	periplasmic solute-binding protein		-5.03
4624	major facilitator superfamily protein	-7.17	-6.24
4665	major facilitator superfamily protein	-9.34	
4689	glutathione transferase	-2.79	
4874	aspartate aminotransferase		-7.76
4903	arylesterase		-3.48
4942	PAS/PAC sensor hybrid histidine kinase	-2.47	
4996	two-component regulatory system, response regulator protein	4.37	
5004	cytochrome c biogenesis protein	6.51	
newgene_133	Type II/IV secretion system secretin, associated with Flp pilus assembly	-4.19	-3.07
